# Predictable NHEJ Insertion and Assessment of HDR Editing Strategies in Plants

**DOI:** 10.3389/fgeed.2022.825236

**Published:** 2022-03-16

**Authors:** Kutubuddin A. Molla, Justin Shih, Matthew S. Wheatley, Yinong Yang

**Affiliations:** ^1^ Department of Plant Pathology and Environmental Microbiology and the Huck Institutes of the Life Sciences, The Pennsylvania State University, University Park, PA, United States; ^2^ ICAR-National Rice Research Institute, Cuttack, India

**Keywords:** CRISPR-Cas9, genome editing, guide RNA engineering, homology-directed repair, non-homologous end joining, NHEJ prediction

## Abstract

Canonical CRISPR-Cas9 genome editing technique has profoundly impacted the fields of plant biology, biotechnology, and crop improvement. Since non-homologous end joining (NHEJ) is usually considered to generate random indels, its high efficiency mutation is generally not pertinent to precise editing. Homology-directed repair (HDR) can mediate precise editing with supplied donor DNA, but it suffers from extreme low efficiency in higher plants. Therefore, precision editing in plants will be facilitated by the ability to predict NHEJ repair outcome and to improve HDR efficiency. Here, we report that NHEJ-mediated single nucleotide insertion at different rice genes is predictable based on DNA sequences at the target loci. Three mutation prediction tools (inDelphi, FORECasT, and SPROUT) have been validated in the rice plant system. We also evaluated the chimeric guide RNA (cgRNA) and Cas9-Retron precISe Parallel Editing via homologY (CRISPEY) strategies to facilitate donor template supply for improving HDR efficiency in *Nicotiana benthamiana* and rice. However, neither cgRNA nor CRISPEY improved plant HDR editing efficiency in this study. Interestingly, our data indicate that tethering of 200–250 nucleotides long sequence to either 5′ or 3′ ends of guide RNA did not significantly affect Cas9 cleavage activity.

## Introduction

Crop improvement greatly relies on exploiting existing- and creating new-genetic variations. Conventional CRISPR-Cas tools have greatly facilitated the generation of targeted genetic variations in plants by producing random indels through the non-homologous end joining (NHEJ) repair pathway (Xie and Yang, 2013; Molla et al., 2020a). Base editing, an emerging technology, can precisely install four transition and two transversion point mutations (Molla and Yang, 2019; Molla and Yang, 2020a; Molla et al., 2020b; Molla et al., 2020c). However, neither CRISPR-Cas nor base editing can generate precise indels, which are also important for plant trait improvement. To generate precise indels, we mainly depend on utilizing the homology-directed repair (HDR) pathway. Unfortunately, HDR is template-directed and inefficient, limiting its application in crop improvement. Cas9-induced double-strand break (DSB) in DNA is repaired predominantly through the NHEJ pathway in higher plants. Therefore, unlike HDR, NHEJ-mediated mutagenesis is highly efficient in plants. If we could predict the DSB repair outcome, it would facilitate generating precise indels.

Although Cas9 was believed to trigger random repair outcomes, a growing body of evidence indicates that the repair outcomes are non-random and depend on target DNA sequence (van Overbeek et al., 2016; Chakrabarti et al., 2019; Taheri-Ghahfarokhi et al., 2018; Molla and Yang, 2020b; Li et al., 2021). Large datasets were utilized to develop models (inDelphi, FORECAST, SPROUT, and CROTON) for predicting Cas9 repair outcome in mammalian cells (Shen et al., 2018; Allen et al., 2019; Leenay et al., 2019; Molla and Yang, 2020b; Li et al., 2021). Those computational tools predict repair outcomes, mainly the frequency of 1 bp insertions and small deletions, with high efficiency (Molla and Yang, 2020b). Although the models have been developed exploiting Cas9 repair data from mammalian cells, we hypothesize that they could also be used to predict repair outcomes in plant cells since the mutations largely depend on the local DNA sequence near the DSB (Molla and Yang, 2020b). However, no single study reports Cas9 repair outcome prediction in plants or validates those predictor models in plants.

HDR is highly valuable in precise gene replacements, knock-in, and installing complex modifications. However, achieving a decent efficiency in higher plants is a major hurdle to using HDR regularly in crop improvements. For HDR to be successful, adequate donor repair templates are needed to be available near the DSB. Temporal and spatial coordination between DSB creation and supplying adequate donor templates are considered the major bottlenecks in HDR (Li et al., 2018; Huang and Puchta, 2019). Several strategies such as using geminivirus replicons (Čermák et al., 2015), chimeric guide RNA (cgRNA) (Butt et al., 2017), chemically modified donor DNA, tandem repeat HDR (TR-HDR) (Lu et al., 2020), and transcript-templated HDR (TT-HDR) (Li et al., 2019) have been used to overcome these bottlenecks. To make the donor templates available onsite of DSB, one attractive approach is to fuse them with the guide RNA (gRNA) sequence. For example, the use of chimeric guide RNA (cgRNA) molecule, containing gRNA fused with donor template, has been demonstrated to induce HDR in rice (Butt et al., 2017). cgRNA strategy was based on donor template fusion at the 3’ end of sgRNA and RNA-templated DNA repair (Butt et al., 2017). Recently, an interesting strategy, Cas9-Retron precISe Parallel Editing via homologY (CRISPEY), described the utilization of bacterial retron to produce single-stranded donor DNA that is tethered with sgRNA (Sharon et al., 2018). CRISPEY strategy has been shown to improve HDR efficiency up to 96% in yeast (Sharon et al., 2018). In human cells, CRISPEY achieved HDR rates of up to 11.3% (Kong et al., 2021; Zhao et al., 2021). However, there is no report of plant HDR improvement utilizing a bacterial retron system.

In this study, we explored to achieve HDR-mediated editing using cgRNA and CRISPEY strategies in tobacco and rice for six different target genes. We also investigated predicting Cas9 repair outcome in plants utilizing the predictor models generated for mammalian systems. The findings could be helpful for precise genome editing in plants.

## Materials and Methods

### Vector Construction

We have designed modified versions of the CRISPEY construct described earlier (Sharon et al., 2018). For GFP to BFP conversion in *Nicotiana benthamiana*, we fused tobacco codon optimized *E. coli* Ec86-reverse transcriptase (Ec86-RT) with P2A-Cas9 for co-translational expression of both Ec86-RT and Cas9 by CaMV35S promoter. P2A is a self-cleaving peptide. A chimeric RNA of Ec86 retron sequence with a gRNA was expressed by AtU6 promoter. It was designed in such a way that a portion of retron sequence was replaced with a donor template sequence harboring the mutations necessary for GFP to BFP conversion (Supplementary Table S1 and Supplementary Sequence).

For rice, we first constructed a basic vector pK-CRISPEY, which contains three distinct cassettes. The first one is a rice codon-optimized Ec86-RT expression cassette. Ec86-RT was driven by OsUbi10 promoter and terminated with Agrobacterium gene seven terminator. The second one was to express a chimeric RNA of Ec86 retron sequence with a gRNA. A portion of retron sequence was replaced with specific donor template sequences. This cassette was driven by CaMV 35S promoter and terminated by Arabidopsis HSP terminator. The 5′ and 3′ end of the chimeric retron-guide sequence was flanked by the hammerhead (HH) ribozyme and the hepatitis delta virus (HDV) ribozyme, respectively. Two Aar1 sites were incorporated upstream of the gRNA scaffold sequence for easy cloning of donor template plus protospacer sequence. The donor template was at the 5’ end of the protospacer. The third cassette was to express SpCas9. We synthesized the first two cassettes and cloned them at the HindIII/BsaI sites of pRGEB32 vector replacing 402 bp to construct pK-CRISPEY. The original hygromycin phosphotransferase (HPT) gene cassette of pRGEB32 was intact (Xie et al., 2015). We separately synthesized donor plus protospacer for each of the three targets, *OsALS*, *OsCC,* and *OsActin,* and cloned at the AarI sites of pK-CRISPEY. Three vectors were named as pK-CRISPEY-ALS, pK-CRISPEY-CC, and pK-CRISPEY-Actin (Supplementary Table S1 and Supplementary Sequence).

For the cgRNA approach, a polycistronic-tRNA-gRNA (PTG) multiplexing cassette was designed to repair three targets simultaneously (Xie et al., 2015). It comprises three gRNAs, *OsALS, OsPita*, and *OsPtr*, each with their specific repair template at the 3′ ends of the scaffolds. *ALS* and *Pita* required only a few base pairs modification, so the repair template was designed with 100 bp homology flanks from the DSB (Supplementary Table S1). Since the *Ptr* needed a 12 bp deletion and various base-pair changes over a larger region, we used 125 bp homology arms. Synonymous mutations were introduced in repair templates to prevent Cas9 from re-cutting after successful HDR repair. The PTG fragment with repair templates was synthesized (GenScript, NJ, United States) and then cloned downstream of the OsU3 promoter into the binary vector pRGEB32 using the compatible overhangs generated by BsaI digestion. Guide RNAs with repair templates fused at their 3’ ends are termed as chimeric guide RNA (cgRNA) following an earlier report (Butt et al., 2017). This new construct is termed pCgAPP (Supplementary Sequence).

pK-CRISPEY-ALS, pK-CRISPEY-CC, pK-CRISPEY-Actin, and pCgAPP were introduced into *Agrobacterium tumefaciens* strain EHA105 *via* electroporation for subsequent agroinfiltration and/or stable transformation in tobacco and rice.

### Agroinfiltration and Generation of Stable Transgenics in *Nicotiana benthamiana*

*Nicotiana benthamiana* 16c, a transgenic line highly expressing mGFP, was used in this study (Ruiz et al., 1998). 16c line was a generous gift from Prof. David Baulcombe (United Kingdom). Agroinfiltration was performed using four to 6 week old plants grown at 25°C and 75% humidity (75%) under the 16 h light (100 µmol photons m^−2^ s^−1^) according to a previously described protocol with few modifications (Yang et al., 2000). Briefly, MMA solution (10 mM MES, 10 mM MgCl2, 150 μM acetosyringone) was used as infiltration solution to resuspend *Agrobacterium* cells (EHA105) to an OD_600_ = 1. The abaxial leaf regions to be infiltrated were punctured with a small needle. A 1-ml syringe (without needle) was used to infiltrate *Agrobacterium* suspension.

To develop stable transgenic plants, fully expanded fresh leaves were collected and sterilized by immersing in 70% ethanol for 60 s, washing in 7.5% bleach plus one drop Tween-20 solution for 20 min, and repeatedly washing in sterile distilled water. Round leaf discs were prepared by pressing a cork borer against an *N. benthamiana* leaf on a Petri dish base. Leaf discs were incubated in *Agrobacterium* suspension (OD_600_ = 1, 100 µM acetosyringone) for 30 min. The discs were blot dried and incubated in cocultivation media (4.3 g/L MS salts, 30 g/L sucrose, 1 mg/L 6-benzylaminopurine, 0.1 mg/L 1-naphthaleneacetic acid, and 100 μM Acetosyringone) for 2 days in the dark. Leaf discs were washed for removing extra bacterial cells, blot dried, and transferred to regeneration selection media (4.3 g/L MS salts, 30 g/L sucrose, 1 mg/L 6-benzylaminopurine, 0.1 mg/L 1-naphthaleneacetic acid, 400 mg/L Timentin, and 10 mg/L hygromycin). The plates were incubated at 28°C with an 18 h light regime. Leaf discs were moved to fresh media plates every 14–15 days. After 2 weeks of selection, callus tissue starts appearing from the cut ends of the disk. Shoots growing from the selected calli were dissected and placed in rooting media (MS salts, 30 gm/L sucrose, 25 mg/L hygromycin) to produce plantlets. Shoots were individually excised from the calli once they reached a height of >3 mm. After 2 weeks in rooting media, roots were adequately developed. Plantlets with well-developed roots were transferred to soil pot in greenhouse.

### Rice Transformation and Regeneration

Kitaake and Jupiter (*Oryza sativa* subsp. *japonica*) rice genotypes was used for genetic transformation. Mature embryo-derived calli were transformed with all four constructs using the *Agrobacterium*-mediated method following an earlier described protocol (Molla et al., 2020a). Briefly, transformed calli were selected in hygromycin (50 mg/L) containing media. Selected and proliferating calli were either transferred to regeneration media or collected for DNA isolation. Regenerated shoots were transferred to rooting media. Well-rooted plantlets were transferred to soil and grown in a greenhouse.

### Microscopical Analysis

Segments of leaf tissue (1–2 cm) were excised, and the pieces were mounted in water on glass microscope slides with a coverslip. The leaves were imaged using an Observer SD spinning disc confocal microscope (Zeiss, Germany). Samples were visualized and photographed using 405 nm (Blue) and 488 nm (Green) filters.

### Genotyping of Editing Outcomes

For the three CRISPEY constructs, hygromycin-resistant calli were used for DNA isolation after two rounds of selection. Isolated DNAs from calli samples were used to amplify the target regions. PCR products were pooled and subjected to deep amplicon sequencing by using Genewiz amplicon EZ sequencing service (Genewiz, United States). After adapter ligation and library preparation, the samples were sequenced using a 2 × 250 paired-end configuration. Image analysis and base calling were conducted by the Illumina Control Software on the Illumina instrument. Raw sequence data were demultiplexed using bcl2fastq version 2.17.1.14. Read pairs were trimmed for adapter sequences and low-quality basecalls using Trimmomatic version 0.36. Each read pair was then merged using the bbmerge tool from the BBtools software toolkit. The target sequence between conserved flanking primers was extracted from each merged pair. For each sample, one excel file was generated to contain the unique nucleotide sequences and their abundances, and one excel file was generated to contain the unique amino acid sequences and their abundance for each sample. The QIIME data analysis package was used to generate OTU sequences. OTU clusters are defined by a 97% identity threshold.

We also regenerated plants (Jupiter variety) for pCgAPP construct. Total DNA was isolated from leaf samples collected from each individual plant following an earlier described protocol (Molla et al., 2020c). Target regions of *ALS*, *Pita*, and *Ptr* loci were amplified by PCR using specific pair of primers. Purified PCR products were sequenced and decoded using TIDE for editing outcomes (Brinkman et al., 2014). All primers used for genotyping are listed in Supplementary Table S1.

### Prediction of NHEJ Outcome

We employed three models, inDelphi, FORECast and SPROUT, for predicting NHEJ mutation outcome (Molla and Yang, 2020b). The inDelphi is available with the link: https://indelphi.giffordlab.mit.edu/. Protospacer with 50 bp flanking sequence on each side was provided as input in the inDelphi user interface. FORECasT was accessed with the link: https://partslab.sanger.ac.uk/FORECasT, while SPROUT was accessible by following the link: https://zou-group.github.io/SPROUT. For FORECasT, around 40–50 bp target genomic sequence, including protospacer, is required. SPROUT requires 20 bp protospacer plus 3 bp PAM sequence for prediction. The prediction outputs from each model were compared with the observed data from deep sequencing (*ALS, CC,* and *Actin* targets) and Sanger sequencing (*Pita* target). The data from retron and cgRNA experiments were reanalyzed for NHEJ outcomes. We have considered the single base pair insertion and different deletion types and their frequency in our analysis.

### Statistical Analysis

The data were analysed using Graphpad prism nine software (GraphPad Software, La Jolla, CA, United States). One-way analyses of variance (ANOVAs) and Dunnett’s multiple comparisons test were used to compare the differences between different groups.

## Results and Discussion

### Utilizing Retron for HDR in *Nicotiana benthamiana* and Rice

**FIGURE 1:**
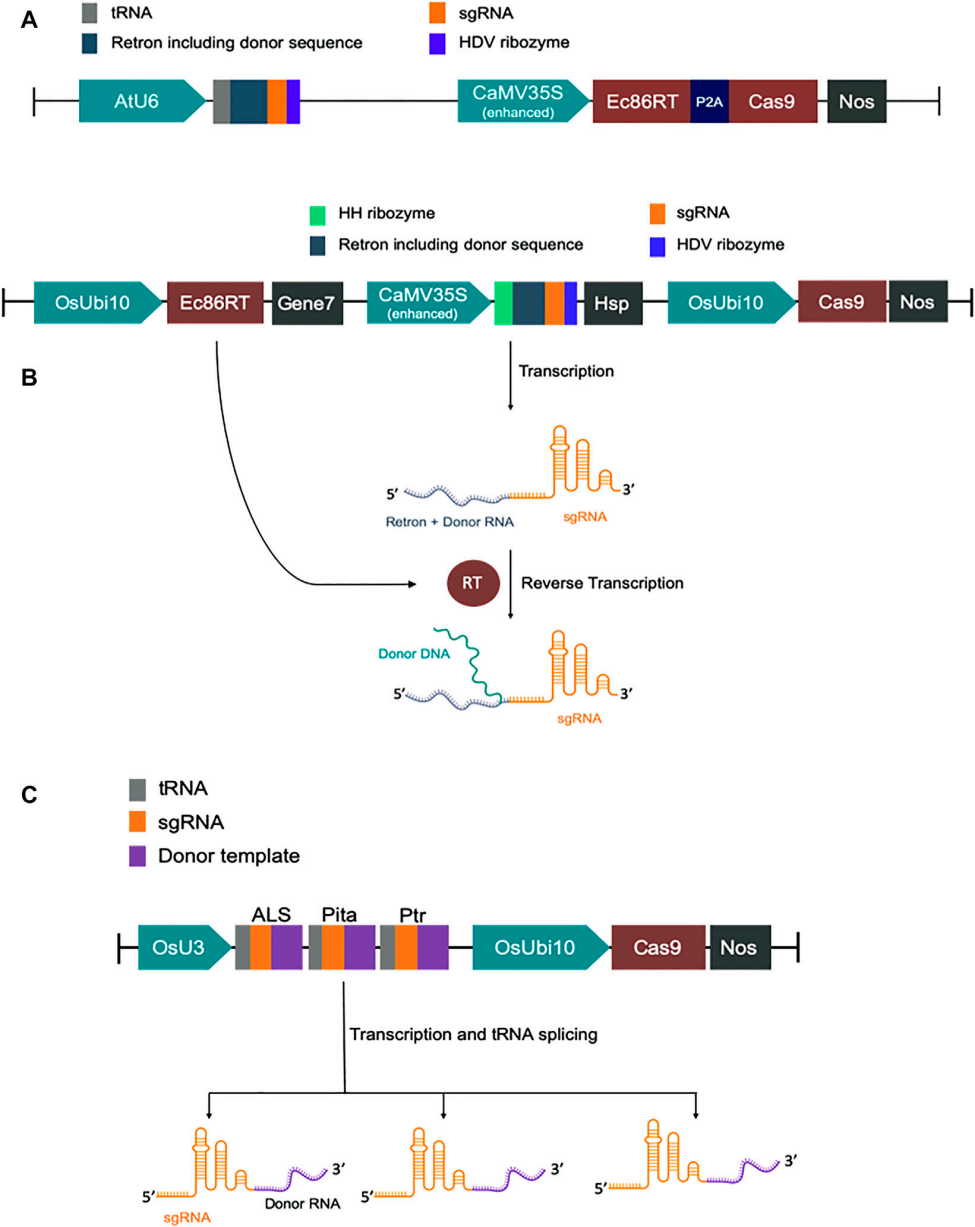
Schematic diagram of DNA constructs. **(A)** Map of CRISPEY constructs used in *Nicotiana benthamiana*. **(B)** Map of pK-CRISPEY with donor used in rice. **(C)** Schematic diagram of pCgAPP construct.

Retrons are prokaryotic retroelements that can produce multicopy single-stranded DNA (msDNA). Bacterial retrons have recently been shown to function in antiphage defense (Millman et al., 2020). Retron Ec86, from *E. coli*, contains a cassette that encodes a unique RNA (msd-msr) and a reverse transcriptase (Ec86-RT) (Inouye et al., 1999). Ec86-RT can reverse transcribe the msd portion into single-stranded DNA that remains tethered to its template RNA (Inouye et al., 1999). By altering a part of msd-msr sequence, single-stranded DNA (ssDNA) containing desired mutations flanked by homology to a targeted genomic region could be produced *in vivo*. Retron-derived ssDNA has been demonstrated to facilitate template-mediated genome editing in yeast (Sharon et al., 2018; Gallagher et al., 2020), mammalian cells (Kong et al., 2021; Zhao et al., 2021), and bacteria (Schubert et al., 2021). We fused the retron seq (msd-msr altered with donor sequence of interest) at the 5′ end of the gRNA to generate a chimeric transcript. After reverse transcription by Ec86RT, the donor ssDNA template would be tethered to the transcript and be available near the DSB for template-dependent repair. Envisaging retron could be harnessed for HDR-editing in plant cells, we set out to validate first in a GFP expressing *N. benthamiana* 16c line*.* To test if retron can promote HDR, we used a reporter system that results in GFP to BFP conversion. We designed a binary construct to express Cas9, Ec86RT, and retron sequence harboring information to produce donor ssDNA templates (Figure 1A). We introduced a single nucleotide change (TAT > CAT) for mGFP to BFP conversion (Tyr > His) and an additional three synonymous mutations in the protospacer seed region to prevent re-cutting the donor template by Cas9. We could not detect blue fluorescence when we analyzed leaf samples by confocal microscopy after several rounds of agroinfiltration. Similar results were obtained for samples collected after 3, 7, and 10 days of infiltration. Assuming that transient expression might not be sufficient to induce HDR, we performed leaf disc transformation with the construct and regenerated >50 stably transformed plants (Supplementary Figures S1A,B). Plants were analyzed for change in fluorescence from green to blue. We could not find a single plant with altered fluorescence (Supplementary Figure S1C). Sanger sequencing of 30 randomly chosen plants also did not reveal successful editing. We reasoned that the failure might be due to the inefficiency of the guide RNA used for the experiment. The guide RNA contains 5′-CTTA-3′ immediately adjacent to PAM sequence (Supplementary Figure 1D). The 5′-CTTA-3′ motif has been shown to be inefficient in genome editing in an earlier systematic study (Graf et al., 2019).

Then we attempted to test the same strategy in rice by targeting three genes, *OsALS* (OsKitaake02g183100), *OsCC* (OsKitaake05g165200), and *OsActin* (OsKitaake03g316400), separately (Figure 1B). For *ALS*, one nucleotide change (TGG > TTG) causing W > L for herbicide tolerance and another nucleotide change for PAM destruction were included in the donor template. We attempted to knock-in a 12 bp sequence harboring EcoRI and HindIII recognition sites in *CC* target and 6 bp EcoRI recognition sequence in *Actin* target. Three constructs were independently transformed to rice calli via the *Agrobacterium*-mediated transformation. After two consecutive selections in hygromycin, proliferating calli were collected for DNA isolation. For initial verification, we amplified the *ALS* target region and digested it with Mfe1 since successful editing events should generate a recognition site for the enzyme. We observed many samples exhibited Mfe1 positive results. It should be noted here that Mfe1 could also be generated if there is a single T insertion (NHEJ) at the cut point. Then, we sequenced randomly chosen 12 samples and analyzed them using Synthego ICE tool. Excitingly, the ICE analysis revealed a knock-in efficiency of up to 23% in the tested calli for the *ALS* target (Supplementary Figure S2).

For further validating the result and obtaining a clear idea of editing efficiency, we performed amplicon deep sequencing. For each construct, DNA was isolated from 30 independent calli samples. We amplified target regions using PCR and pooled 10 samples into one for deep amplicon sequencing. On an average, we obtained >50 K reads for each sample. Strikingly, results revealed no HDR events occurred in the case of *ALS* and *Actin* targets. This observation indicated that, for one or two nucleotide replacements, as in the case of *ALS*, the ICE tool is not highly sensitive and may mislead on the editing output. Amplicon deep sequence is recommended to get a clear picture of the editing events. However, for the *CC* target, we observed only one read with a perfect 12 bp knock-in. We have observed high NHEJ efficiency in all three cases. Overall, retron-mediated template editing has not been successful in tobacco and rice. Around 16 bacterial retron systems have been experimentally validated and have their fully annotated components available in the public database (Simon et al., 2019). Recently, modifications in the retron non-coding RNA that increases production of reverse transcribed DNA have been identified (Lopez et al., 2021). Systematic studies are required to find suitable retron systems for plant genome editing.

### Chimeric gRNA Approach for HDR in Rice

**FIGURE 2:**
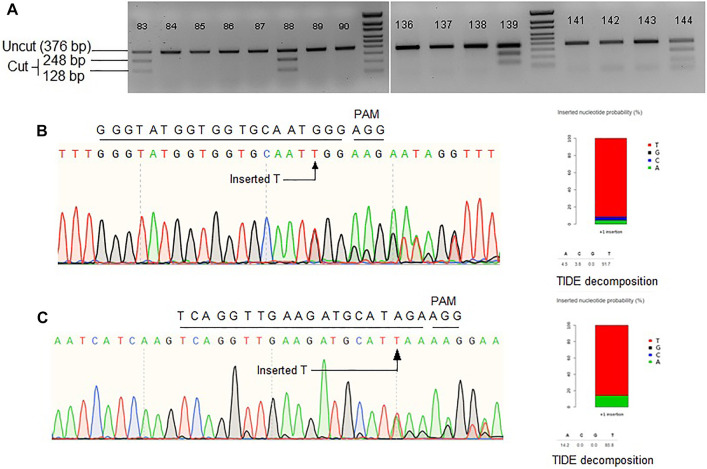
**(A)** Representative gel images showing Mfe1 digestion of *ALS* product. Single T insertion at the cut point generates Mfe1 recognition site. **(B)** Representative Sanger chromatogram of *ALS* locus. **(C)** Representative Sanger chromatogram of *Pita* locus. TIDE decomposition of the chromatogram in **(B,C)** showing single T insertion. Wild-type protospacer and PAM sequences are shown above each of the chromatograms.

Another approach to make the repair template available at the vicinity of DSB is to fuse the template sequence with gRNA sequence. After successful transcription, a chimeric guide RNA (cgRNA) would be produced containing gRNA for Cas9-mediated targeted DSB generation and RNA repair template for HDR editing (Figure 1C). An earlier study demonstrated 2.14% HDR efficiency of cgRNA approach in regenerated rice plants (Butt et al., 2017). Similarly, Cas12a mediated DSB coupled with RNA donor template was reported to achieve successful HDR in rice (Li et al., 2019). Encouraged by these studies, we tested the strategy at three rice loci, *ALS*, *Pita*, and *Ptr*. The pCgAPP construct was transformed in rice calli and 196 hygromycin resistant T_0_ rice lines were obtained through regeneration. For the *ALS* and *Pita* targets, successful HDR event would generate the recognition sites for MfeI and NcoI, respectively. To screen successful HDR events at the *Ptr* locus, PCR positive/negative assay was employed by using one primer for the genomic region outside the homology included in repair template and a primer for the region over the deleted 12 bp polymorphism only found in the repair template (Supplementary Figure S3A). For *ALS* target, 25 out of the 196 lines indicated success of the MfeI site generation (Figure 2A). However, no *Nco*I positive lines were generated for *Pita* and no amplification was observed for *Ptr*. These results indicated unsuccessful HDR events at *Pita* and *Ptr* loci.

To further investigate editing outcomes, the lines in which successful MfeI sites generated were sequenced for all three target loci. Sanger chromatograms were decoded, aligned and compared to the WT and the repair template to analyze editing outcomes (Figures 2B,C). While for *ALS* there were many positive MfeI digestion results, sequencing revealed these occurred by single-T insertions (NHEJ indel) and not by HDR. If HDR created the desired changes, no indel should be observed, and all the substitutions from the repair template would be included. It is also interesting that some alleles contain a correct substitution but at one side of the DSB point, suggesting one-sided HDR events (Supplementary Figure S3B). Overall, we observed 12% (3/26) of the alleles to have a possible one-sided HDR in *ALS* by evidence of base substitution. For the *Pita* gene, randomly chosen 17 lines were sequenced. We observed 76% of the lines with indels. We could not detect any instances of HDR in *Pita* locus. Lastly, we sequenced *Ptr* locus in 20 random lines and have not found any evidence of editing either by NHEJ or HDR. The *Ptr* protospacer contained a GCC motif in the first four nucleotides proximal to PAM, which was reported to be inefficient in cleaving by Cas9 (Graf et al., 2019). Based on the results we obtained for the three loci in rice, it seems cgRNA approach is not efficient in mediating HDR editing.

### Prediction of NHEJ-Mediated Precise Insertion in Rice Genes

Cas9 induced DSB generation and subsequent NHEJ-mediated genome editing is highly efficient in plants. The NHEJ repair outcome is considered random and, therefore, not useful in precise genome editing applications. However, several recent studies in animal systems showed that the Cas9-mediated editing outcome is reproducible and predictable depending on the features of target DNA sequences (Molla and Yang, 2020b). The ability to predict the spectrum of DSB repair outcomes would facilitate us in performing more efficient gene knock-out and precision genome editing applications without HDR. Using the DSB repair products of thousands of target DNA loci in mammalian cells, several machine learning models have been generated to predict the spectrum of CRISPR-Cas9 editing products (Shen et al., 2018; Allen et al., 2019; Leenay et al., 2019; Li et al., 2021). To the best of our knowledge, no study has reported predictability of Cas9-induced DSB repair outcome in plants. Therefore, we set out to analyze our dataset to examine if the same prediction rules are applicable in plants and if the existing machine learning models could be applicable to foresee the Cas9-induced mutation outcome in plants.

**FIGURE 3:**
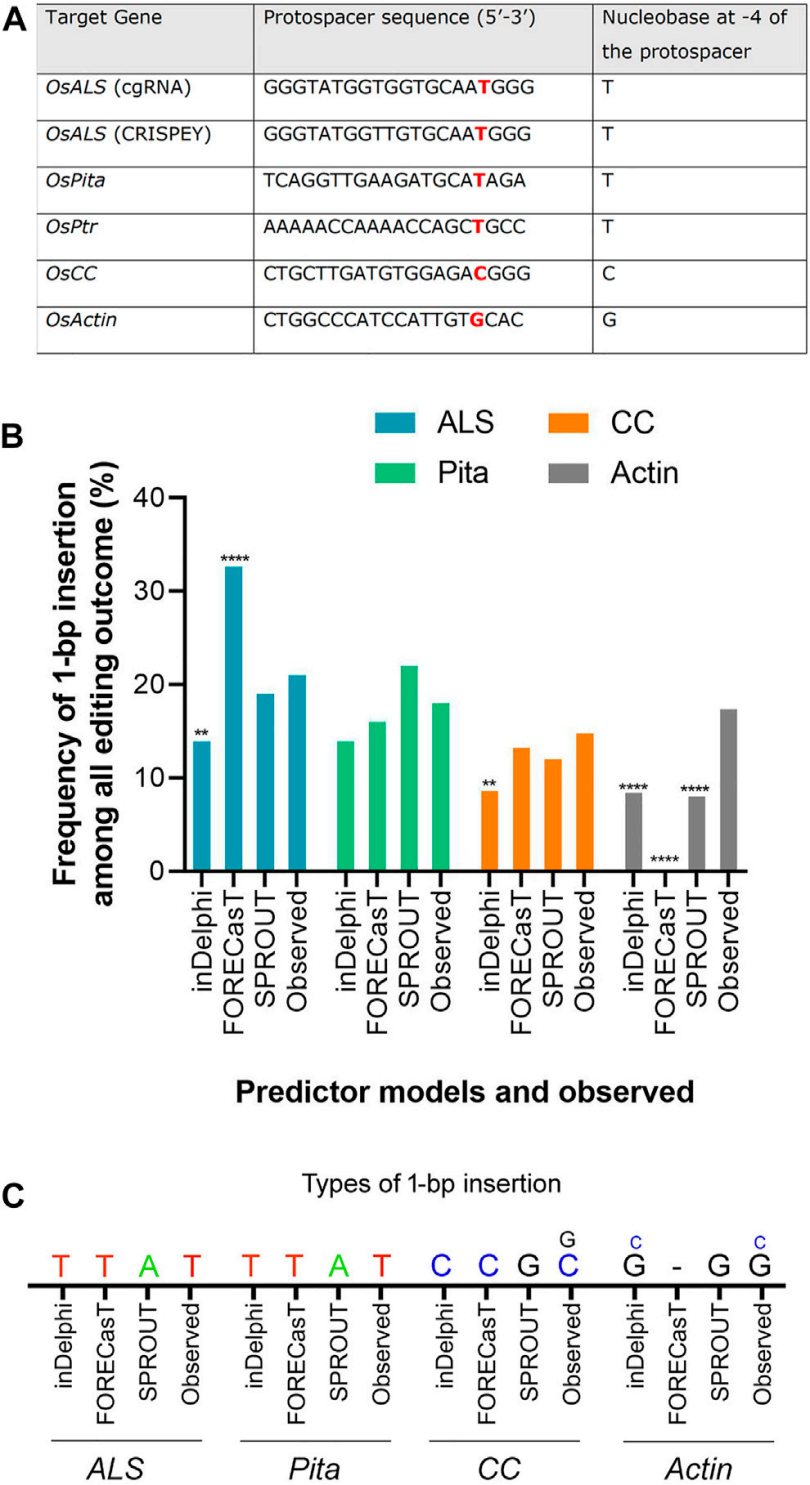
Prediction of 1-bp insertion using models. **(A)** Protospacer sequences used in the study. ALS protospacers for Jupiter (used in cgRNA) and Kitaake (used in CRISPEY) are differed by a single nucleotide. **(B)** Frequency of 1-bp insertion predicted by inDelphi, FORECasT, and SPROUT versus observed frequency. Each prediction data was compared with the observed data. For *ALS*, *CC*, and *Actin* loci, deep sequencing data was treated as observed data. Observed data for *Pita* derived from Sanger sequencing. (**) denotes *p* ≤ 0.01; (****) denotes *p* ≤ 0.0001. **(C)** Types of 1-bp insertion predicted and observed across four loci. Smaller letters indicate second most prevalent insertion.

The editing outcome that could be most reliably predicted is single nucleotide insertion (Molla and Yang, 2020b). Earlier studies reported that the inserted nucleotide is identical to the nucleotide at –4 from the PAM sequence (Chakrabarti et al., 2019; Lemos et al., 2018). Therefore, if a T nucleotide is present at –4 of the protospacer sequence, another T nucleotide is highly likely to be inserted (Chakrabarti et al., 2019). However, the predictability decreases in the order T > A > C > G at –4 position (Molla and Yang, 2020b). For a preliminary investigation, we have carefully chosen three protospacers, for targeting *ALS*, *Pita* and *Ptr*, having a T at –4 position (Figure 3A). The inDelphi model predicted single T insertion for 13.9% of all products in the *ALS* target locus (Figure 3B; Supplementary Figure S4). Interestingly, we observed 25 lines (12.75%) showed positive Mfe1 digestion out of 196 plants tested from our cgRNA experiment, indicating a single T insertion at the cut point. Sanger sequencing of those lines validated the result (Figure 2B). Similarly, deep sequencing data from our retron experiment showed an average of 21.13% single T insertion with the same *ALS* guide (Figure 3B). The FORECasT has also predicted single T insertion as the most dominant class of mutations (Supplementary Figure S4). Although SPROUT predicted 19% of the total reads with insertion, it failed to accurately predict the most likely inserted base (Supplementary Figure S4). For the *Pita* guide, inDelphi and FORECasT computed 13.9% and 16% single T insertion, respectively (Supplementary Figure S5). Our experimental data showed 18% of the *Pita* alleles were with a single T inserted. This time also, SPROUT was inaccurate in predicting the inserted base.

FORECasT, SPROUT, and inDelphi predicted 13.2%, 12%, and 8.6% 1 bp insertion in the *OsCC* target locus, respectively. Our deep sequencing data showed 14.77% single C insertion (Figure 3C). inDelphi and FORECasT accurately predicted a C insertion (Supplementary Figure S6). For the *OsActin* target, our result showed most abundant insertion type is single G (13.76%) followed by single C (3.62%) (Figure 3C). Surprisingly, inDelphi predicted the insertion types accurately. SPROUT displayed that the most likely inserted base is G (Supplementary Figure S7). However, FORECasT was not able to predict the insertions.

**FIGURE 4:**
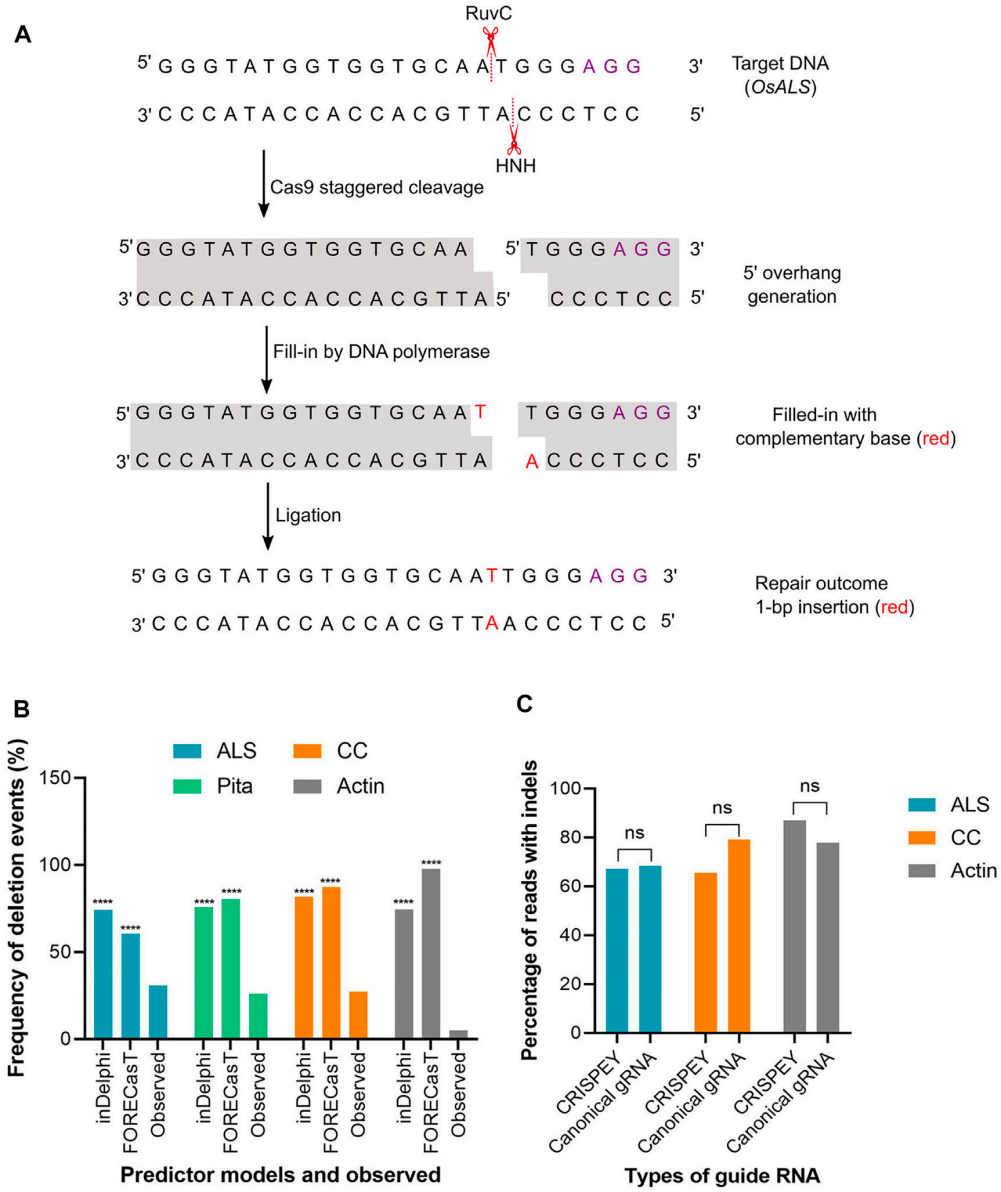
**(A)** A hypothetical model to explain the generation of 1-bp insertion. Os*ALS* target sequence is used as an example. Violet font depicts PAM sequence. Model was redrawn from Molla and Yang (2020b). **(B)** Percentage of deletion events predicted by inDelphi or FORECasT and experimentally observed. Observed value was compared separately with inDelphi and FORECasT predicted values. **(C)** Indels generated with canonical and 5′ extended guide RNA. A 228 bp long sequence was fused at the 5′ end of the gRNA. (****) denotes *p* < 0.0001. ns, non-significant.

The above result indicates that the models are pretty good in predicting the insertion types, especially 1 bp insertion and their fraction in the plant system. We found that the inDelphi outperformed the FORECasT and SPROUT in anticipating the mutation outcome, especially the insertion class and frequency. In contrast to the notion that the NHEJ outcome is random, our data suggest that the Cas9-induced double-strand break repair outcome is non-random and could be predicted (Molla and Yang, 2020b). Single nucleotide insertion is the most predictable class of repair genotype. We found that the inserted nucleotide is identical to the nucleotide at –4 from the PAM sequence in accordance with earlier studies in mammalian cells (Allen et al., 2019; Chakrabarti et al., 2019). The insertion of a single base identical to –4 nucleotide in the protospacer indicates the occurrence of the following sequential events: Cas9-induced 5’ single-base overhang generation, filling in by DNA polymerase, and ligation by ligase 4 (Zuo and Liu, 2016; Lemos et al., 2018; Molla and Yang, 2020b). These events logically explain the generation of 1 bp insertion identical to the –4 base in the noncomplementary strand since the base at –4 acts as a template (Figure 4A). A recent study in mammalian cells showed that the fraction of 1 bp insertion relative to other repair genotypes can be increased by the exogenous application of the ATM kinase inhibitor KU-60019 (Bermudez-Cabrera et al., 2021).

### Prediction of Deletion and Base Substitution

Unlike insertion, the models tested here performed poorly in predicting the frequency and types of deletion and substitution events. Microhomology-mediated end joining (MMEJ) pathway (also known as alternative-NHEJ) is often associated with deletions events generated from Cas9-induced DSB repair. MMEJ deletes intervening bases between short tracts of local matching sequences (Her and Bunting, 2018) and, hence, the repair outcome is predictable by analyzing microhomologous sequences. Since SPROUT does not display deletion types and their respective frequencies, we considered analyzing only inDelphi and FORECasT in this section. Both inDelphi and FORECasT displayed prediction of MMEJ deletion with a high percentage. The difference between the predicted frequency and the observed frequency of deletion events was highly significant (*p* < 0.0001) (Figure 4B). We have also noticed that the models predicted deletions deviated from the observed deletion types in the tested genomic sites (Supplementary Tables S2–4). For example, a AA deletion was found to be the most frequent (average 6.7%) deletion type in the *ALS* locus (Supplementary Table S2). However, inDelphi and FORECasT failed to predict this deletion in their top nine deletion types (Supplementary Figure S4). For *Actin*, both inDelphi and FOREcasT projected an identical 7 bp deletion with >10% frequency (top deletion class) (Supplementary Figure S7). Surprisingly, we could not find a single read with the −7 bp deletion in a total of 179 K reads (Supplementary Table S3). Similarly, observed deletion types at the *CC* locus did not match the predicted deletions (Supplementary Figure S6 and Supplementary Table S4). It is notable that while types of insertion were common across different pooled samples for the same target, deletion types were found rarely common (Supplementary Tables S2–4). In our deep sequencing data, we found a significant amount of reads with combined insertion and deletion. Base substitutions were also common. However, the models (inDelphi, FORECasT, and SPROUT) cannot foresee these kinds of mutation classes. Our results indicate that these models are not good at predicting deletions and base changes in rice plant.

### 5′ and 3’ Modifications of Guide RNA Do Not Seem to Impact CRISPR-Cas9 Editing Efficiency

The commonly used single guide RNA (gRNA) is a fusion of the crispr RNA (crRNA) and transactivating crRNA (tracrRNA) through a short RNA loop (Jinek et al., 2012). Engineering and manipulation of gRNA has been one of the important areas of research for broadening the applications of the CRISPR-Cas system. The introduction of extra nucleotides at either of the gRNA ends could be useful for many genome editing applications, such as adding RNA aptamers to recruit different effector proteins and fusing donor templates for HDR. The degree of gRNA modifications that can be tolerated without affecting its binding with Cas9 and subsequent genome targeting is crucial to define (Nowak et al., 2016). Whether fusion of long sequence at either of the 5′ and 3’ ends of gRNA has any impact in Cas9 cleavage efficiency is not well established in the plant system.

In the retron approach, donor template coding sequences were fused at the 5′ end of the gRNA (Figure 1B). The length of the sequence fused was 228 nucleotides, including the chimeric retron-donor. The 5′ end is crucial for gRNA function as the 20 bp protospacer that determines the genomic target site is located at this end. From our deep sequencing data at all three target sites, it is clear that the DSB formation ability of Cas9 was not hampered at all by the fusion at 5′ end of gRNA. For example, in *ALS*, the CRISPEY construct yielded an average of 67.24% mutation, while the control construct with canonical gRNA showed 68.30% of the population with mutation (Supplementary Table S2). Similarly, for the *Actin* target, 77.86% mutant population was obtained with canonical gRNA, whereas 87% mutant population was generated by 5′ extended gRNA (Supplementary Table S3). *CC* target was mutated with an average efficiency of 79.15% with 5′ extended gRNA (Supplementary Table S4). The results of this study indicate that 5′ extension of gRNA is probably not an inhibitory factor for Cas9 cleavage in plants (Figure 4C). However, a recent *in vitro* study showed that 5′ addition of only two to three unpaired nucleotides in SpCas9 gRNA has a significant effect on the cleavage activity of the RuvC domain (Mullally et al., 2020). Another study reported 5’ end modifications of gRNA retain cleavage activity in mammalian cells, although found some length effect (Kocak et al., 2019).

On the contrary to the retron approach, we fused 200–250 bp donor template sequence at the 3′ end of the respective gRNA sequence (Figure 1C). As evidenced by our result at *ALS* and *Pita* loci, DSB induction efficiency was very high with the gRNA extended at 3′ end. In terms of the overall NHEJ editing efficiencies of the randomly chosen *ALS* lines tested, 92% of the lines had editing, with 84.6% as monoallelic and 7.7% as biallelic. On the other hand, 76.4% of the tested lines for *Pita* had editing, with 47% as monoallelic and 29.4% as biallelic. Of the nine lines where both *ALS* and *Pita* were sequenced, 55.5% (5) displayed editing in both loci, confirming multiplex editing (Supplementary Table S5). These results indicate a fusion of ∼200 nt long sequence at the 3′ end of gRNA did not significantly impact cleavage efficiency in rice *ALS* and *Pita* loci. cgRNAs were also found to be fully functional in generating DSB in rice in an earlier study (Butt et al., 2017). The recently developed prime editing technique depends on a 3′ extended guide RNA (Anzalone et al., 2019). Similarly, modification of the 3′ end of gRNA was well tolerated by SpCas9 in a previous study (Palumbo et al., 2020). Taken together, our data suggest that both 5′ and 3’ ends of gRNA are amenable for modification without significantly affecting the Cas9 cleavage activity in rice.

## Conclusion

We demonstrated that the Cas9 repair outcome, specifically the type and fraction of 1 bp insertion, is predictable in the plant system by employing machine learning models. Among the models tested, inDelphi outperformed the other two models, FORECasT and SPROUT. Applicability of those models to the plant system greatly enhances the ability of plant researchers to better design their experiments for knockout as well as precise genome editing. However, the models failed to accurately predict deletions. We also presented data showing ineffectiveness of retron- or cgRNA-mediated approaches to achieve HDR in rice. Moreover, we showed 5′ and 3′ extension of gRNA with 200–230 nt long sequences did not impact high cleavage activity of Cas9. Although more genomic sites need to be tested for getting a comprehensive idea about the impact of this fusion on DSB generation efficiency, our data would encourage researchers to explore new enhancements to CRISPR-Cas tools.

## Data Availability

The datasets presented in this study can be found in online repositories. The names of the repository/repositories and accession number(s) can be found below: Original raw paired-end sequence data are available in NCBI data base with BioProject accession number PRJNA795336.
