# Supplementary material for: Predictable NHEJ Insertion and Assessment of HDR Editing Strategies in Plants
Source: Front Genome Ed. 2022 Mar 16;4:825236. doi: 10.3389/fgeed.2022.825236 (PMC9037586; doi:10.3389/fgeed.2022.825236)
Supplement: Supplementary file 3 [file Table1.docx]

**Supplementary Table 1: guideRNA sequence, donor template sequence, intended changes incorporated in the donor sequences, and genotyping primer details**

| **Target gene** | **Protospacer (5’-3’)** | **Intended modifications** | **Donor template**  **(Changes are highlighted with red)** | **Genotyping primers** | **Primers’ purpose** |
| --- | --- | --- | --- | --- | --- |
| *mGFP* | CTTGTCACTACTTTCTCTTA | Four bases replacement | aacttacccttaaatttatttgcactactggaaaactacctgttccatggccaacacttgtcactactttTAGtCatggtgttcaatgcttttcaagatacccagatcatatgaagcggcacgacttcttcaagagcgccatgcc | GFP-F: GGTGATGTTAATGGGCACAA  GFP-R: GCCATGATGTATACGTTGTGGGAG | Screening of regenerated *N. benthamiana* plants |
| *OsALS* | GGGTATGGTGGTGCAATGGG  (In Jupiter) | **pCgAPP🡪**  Four base pairs replacement | cccttagcaatagtcacaaaatctggatatatctcgctctcacattccgggttgcccaagtatgtatgcgccctattcgccttgtaaaacctatcTtccAattgGacGaccatacccaaatgttggttgttcaacaccatcaccttcacagggaggttctcaatgcggatcaatgccagctcctgaatgttcatgaggaa | 2211-cgALS-F:  GGCGGCACAATATTACACCT  2212 cgALS-R:  TGTTACACGGACTGCAGGAA | Screening of regenerated rice plants |
|  | GGGTATGGTTGTGCAATGGG  (In Kitaake) | **pK-CRISPEY🡪**  Two base pairs replacement | ggtgaaggtgatggtgttgaacaaccaacatttgggtatggttgtgcaatTggaAgataggttttacaaggcaaatagggcgcatacatacttgggcaac | 2258-ALS-F:  ATCGCTACTGGTGTTGGACA  2259-ALS-R:  CCTGGTGTGGGACGATGATA | Initial PCR for Mfe1-based screening. |
|  |  |  |  | 2377-ALS-Deep-F  ACACTCTTTCCCTACACGACGCTCTTCCGATCTATTGATCCGCATTGAGAACC  2378-ALS-Deep-R  GACTGGAGTTCAGACGTGTGCTCTTCCGATCTTGTTACACGGACTGCAGGAA | Deep amplicon sequencing |
| *OsCC* | CTGCTTGATGTGGAGACGGG | 12 base pairs knock-in | tctgtgctgactgcggggggatggggatctgggtgggggttcgctacgattctctgcttgatgtggagacGAATTCAAGCTTgggcggttgagatcggaacttgttggtgatagatctgcctgtatgcttcgtgttgcatt | 2381-CC-Deep-F:  ACACTCTTTCCCTACACGACGCTCTTCCGATCTCCTTTCTCTGTGCTGACTGC  2382-CC-Deep-R:  GACTGGAGTTCAGACGTGTGCTCTTCCGATCTTAATGCCATATCCCGCAAAT | Deep amplicon sequencing |
| *OsActin* | CTGGCCCATCCATTGTGCAC | 6 bp knock-in | gcttacaatgttgcttgccgttgcagatgtggattgccaaggctgagtacgacgagtctggcccatccattgtgGAATTCcacaggaaatgcttctaattcttcggacccaagaatgctaagccaagaggagctgttatcgccgtcctcc | 2379-Actin-Deep-F:  ACACTCTTTCCCTACACGACGCTCTTCCGATCTGCATCTCTCAGCACATTCCA  2380-Actin-Deep-R:  GACTGGAGTTCAGACGTGTGCTCTTCCGATCTGCGATAACAGCTCCTCTTGG | Deep amplicon sequencing |
| *OsPita* | TCAGGTTGAAGATGCATAGA | 6 base pairs replacement | gatgatcacgggtatggatttttcattctattcccaggttacaacttacaaggattattgagcttctttctttctctgccAtggcttctatctttacctGcGatgcaCTtGcaacctgacttgatgattgtttgaaaccaattttaatggaagttaaatgttattgttgtgaccctgaatcaggttttgtatgctaccgg | 2213 cgPita-F:  GGAGTCTGCCTTGAGGACTG  2214 cgPita-R:  CGTGAAGAGGATTCCGGTAG | Screening of regenerated rice plants |
| *OsPtr* | AAAAACCAAAACCAGCTGCC | 14 base pairs replacement (red) and 12 bp deletion (shown as blue down arrow 🡫) | cggagaaagtacaaattagggtgttatcaagagatacaacacgcgttgggatcttcctcgAccaaaaacataTagtggggcggggGgtatttcgcagGcaTaaactcgtacGgttccggcTtggcagGtggttCA🡫GGCAtaaattcgatgcgatgaacttttacagcttcgttgatgaggtctgcgaaggtcatgcgaggcttcgttgtctggtcgcagataccagcggcgatgttgttgaaccgagcag | 2145 Ptr-HDR-mut-F:  AATTTATGCCTGAACCACCT  2144 Ptr-HDR-R  ACGCGTTGGGATCTTCCTCG | Screening of regenerated rice plants |
