# Supplementary material for: Predictable NHEJ Insertion and Assessment of HDR Editing Strategies in Plants
Source: Front Genome Ed. 2022 Mar 16;4:825236. doi: 10.3389/fgeed.2022.825236 (PMC9037586; doi:10.3389/fgeed.2022.825236)
Supplement: Supplementary file 4 [file Table5.DOCX]

| **Types of editing** | **Loci** | | |
| --- | --- | --- | --- |
|  | **ALS** | **Pita** | **Ptr** |
| Mono-allelic editing  (lines) | 11/13  (84.6%) | 8/17  (47%) | - |
| Bi-allelic  Editing (lines) | 1/13  (7.7%) | 5/17  (29.4%) | - |
| Any Editing  (lines) | 12/13  (92%) | 13/17  (76.4%) | - |
| No editing (lines) | 1/13  (8%) | 4/17  (23.5%) | 20/20 |
| Multiplexed Lines | 5/9  (55.5%) | | |

**Supplementary Table 5:** Details of genotyping (by Sanger sequencing) of plants from the cgRNA experiment
