# Supplementary material for: Predictable NHEJ Insertion and Assessment of HDR Editing Strategies in Plants
Source: Front Genome Ed. 2022 Mar 16;4:825236. doi: 10.3389/fgeed.2022.825236 (PMC9037586; doi:10.3389/fgeed.2022.825236)
Supplement: Supplementary file 6 [file DataSheet1.DOCX]

**Predictable NHEJ insertion and assessment of HDR editing strategies in plants**

Kutubuddin A. Molla^1,2†,^, Justin Shih^1†^, Matthew Wheatley^1^, and Yinong Yang^1*^

^1^Department of Plant Pathology and Environmental Microbiology and the Huck Institutes of the Life Sciences, The Pennsylvania State University, University Park, PA 16802, USA

^2^ICAR- National Rice Research Institute, Cuttack-753006, Odisha, India

**SUPPLEMENTARY INFORMATION**

**Supplementary Figure 1:** CRISPEY (GFP-BFP) experiment in *Nicotiana benthamiana*

**Supplementary Figure 2:** Analysis of Sanger chromatogram with Synthego ICE tool

**Supplementary Figure 3: A,** PCR assay used for genotyping at *OsPtr* locus. **B,** Schematic to show possible one-sided HDR observed at *OsALS* locus.

**Supplementary Figure 4:** Representative screenshot showing prediction of Cas9 repair outcome at the *ALS* locus.

**Supplementary Figure 5:** Representative screenshot showing prediction of Cas9 repair outcome at the *Pita* locus.

**Supplementary Figure 6:** Representative screenshot showing prediction of Cas9 repair outcome at the *CC* locus.

**Supplementary Figure 7:** Representative screenshot showing prediction of Cas9 repair outcome at the *Actin* locus.

**Supplementary Sequences:** Annotated nucleotide sequence for constructs used in this study


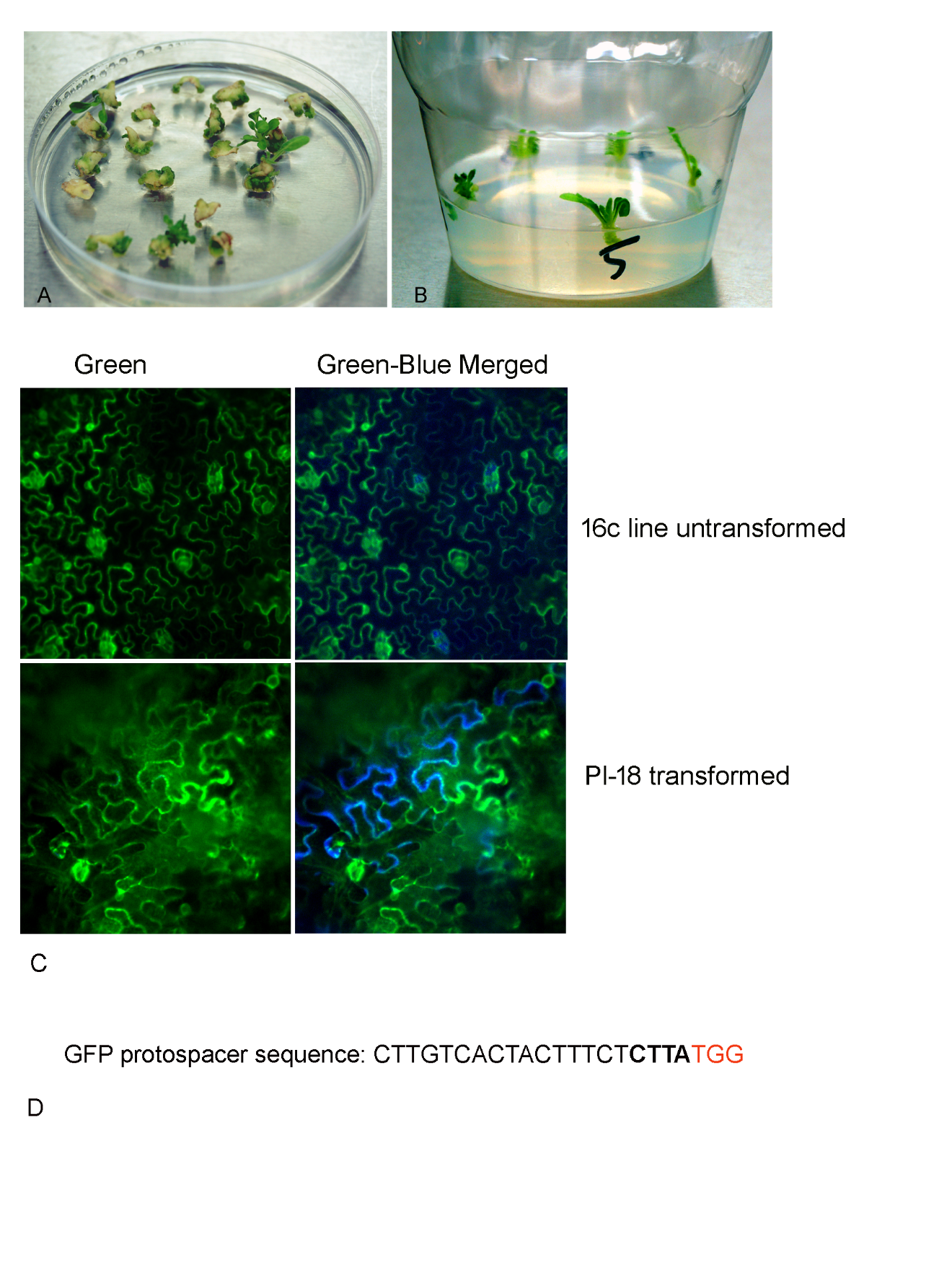


**Supplementary Figure 1:** A. *Nicotiana benthamiana* leaf disc transformed with CRISPEY construct (for GFP-BFP) in regeneration cum selection media. B. Selected seedling in rooting media. C. Confocal microscopy of regenerated plants. Visualized in 405 nm (Blue) and 488 nm (Green) filter. 16c, constitutively GFP expressing *N. benthamiana* line; Pl-18, Plant line 18 transformed with CRISPEY GFP-BFP construct. D. The 20 bp protospacer sequence used in the experiment. PAM is shown in red color. The motif CTTA (bold) is reported to be inhibitory to cleavage efficiency.


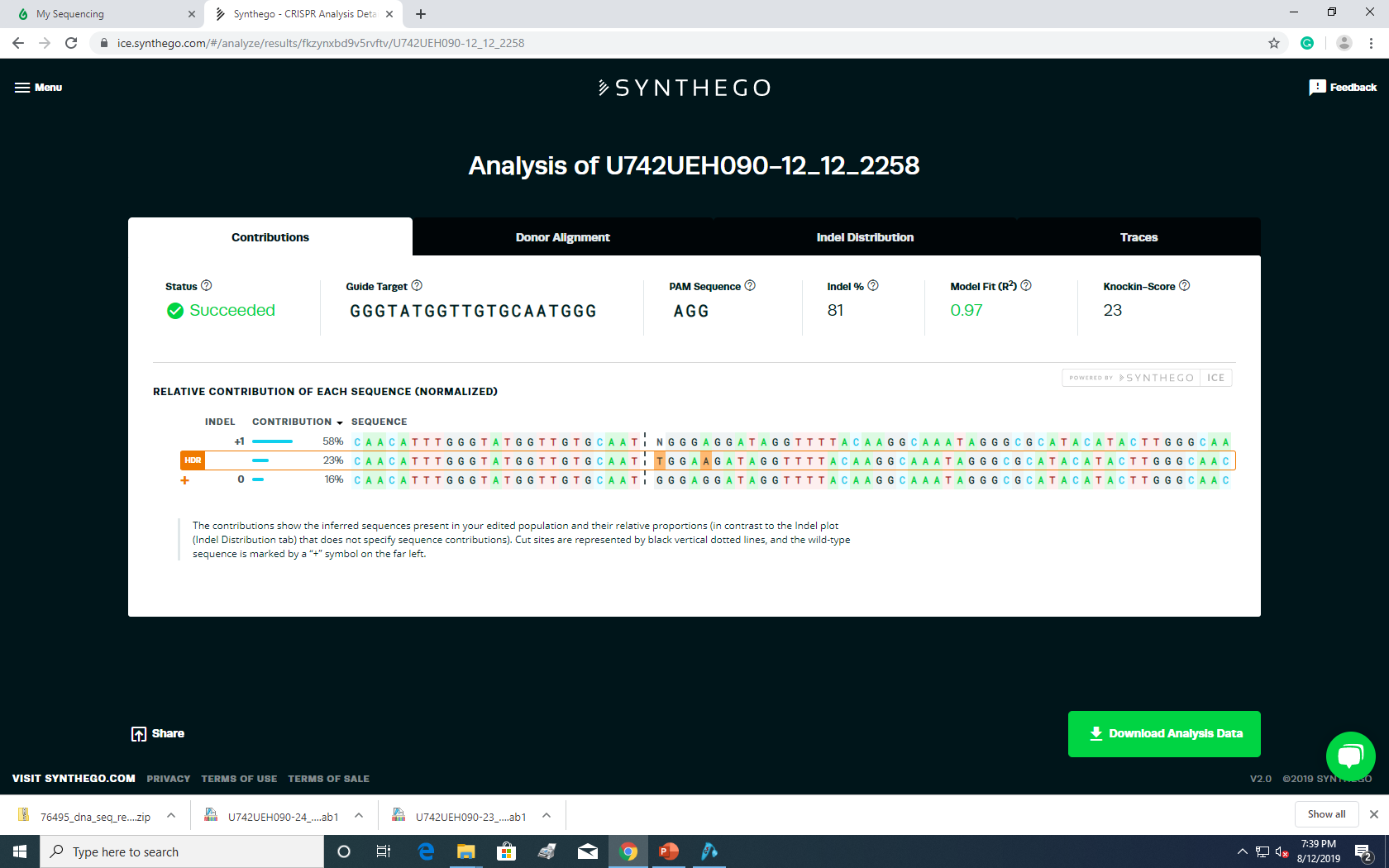


**Supplementary Figure 2:** Representive screenshot showing analysis of Sanger chromatogram with Synthego ICE tool. Analysis showed highest knockin score (HDR efficiency) of 23.


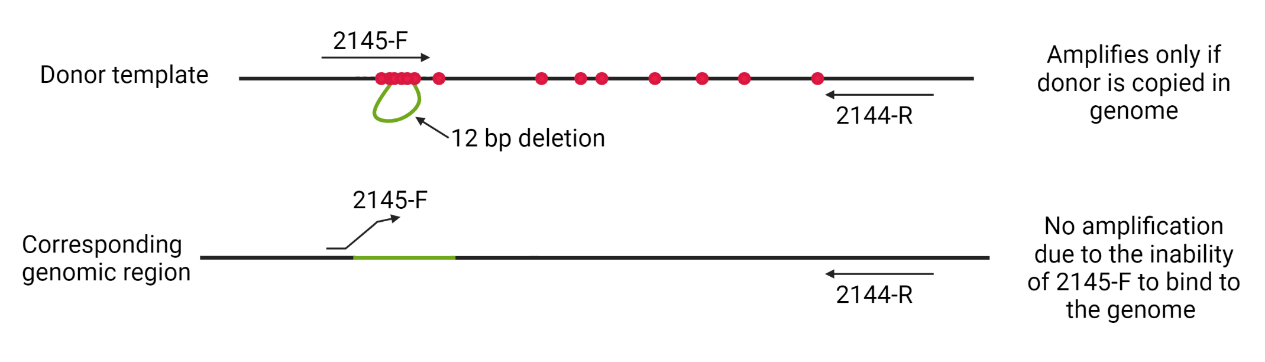

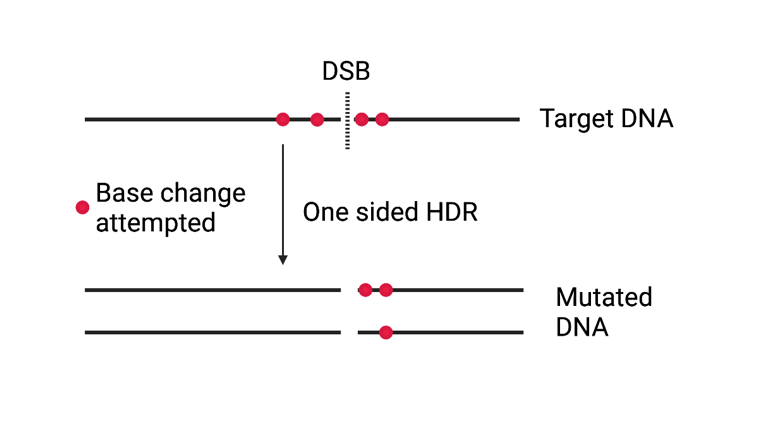


A

B

**Supplementary Figure 3:** **A,** PCR assays for genotyping at the *OsPtr* locus. Magenta dots represent SNP changes. Green bubble shows deleted region in the donor template. Primer 2145-F selectively bind to only donor template encoded sequence. **B,** Schematic to show possible one-sided HDR we observed at *ALS* locus.


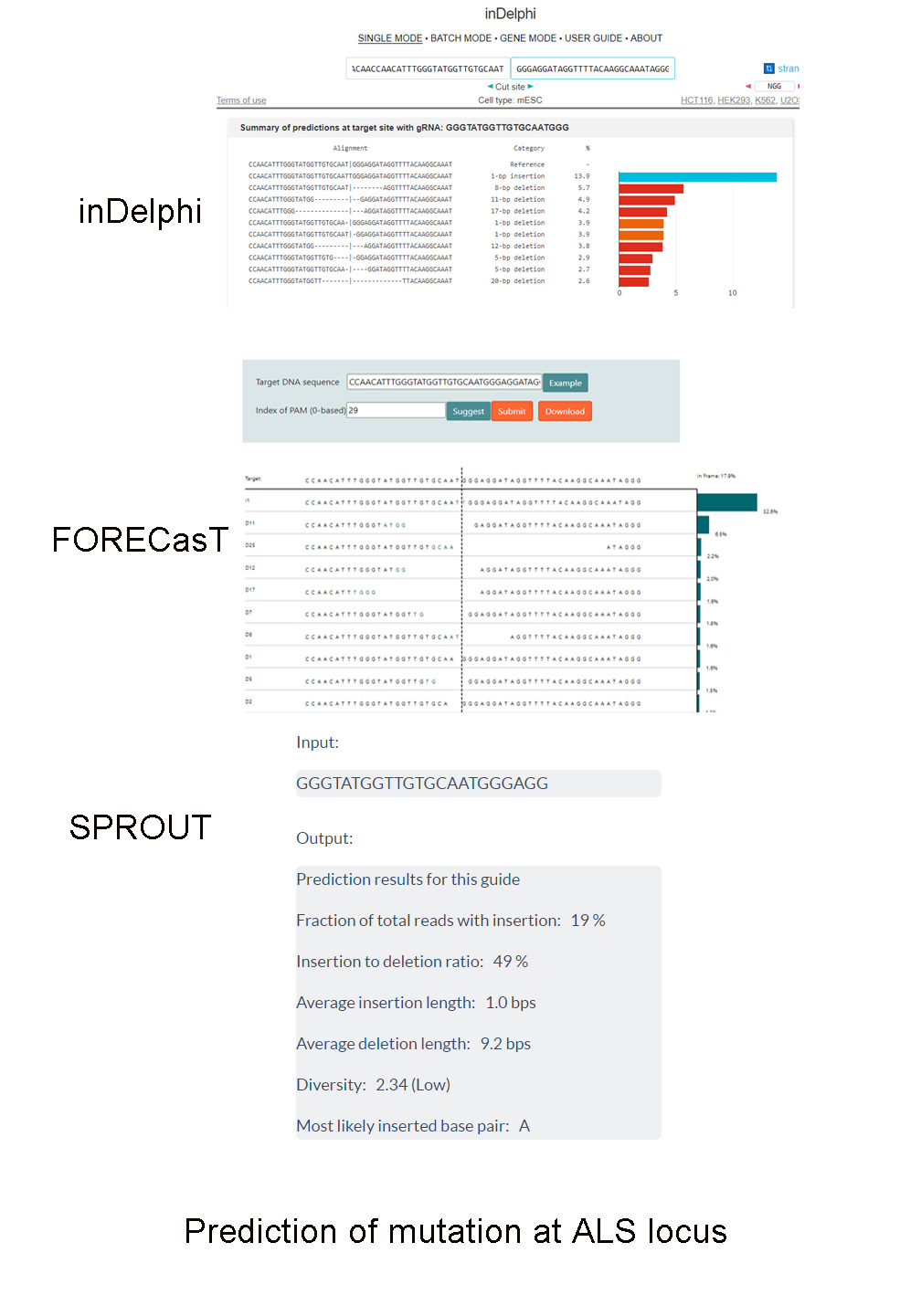


**Supplementary Figure 4:** Representative screenshot showing prediction of Cas9 repair outcome at the *ALS* locus.


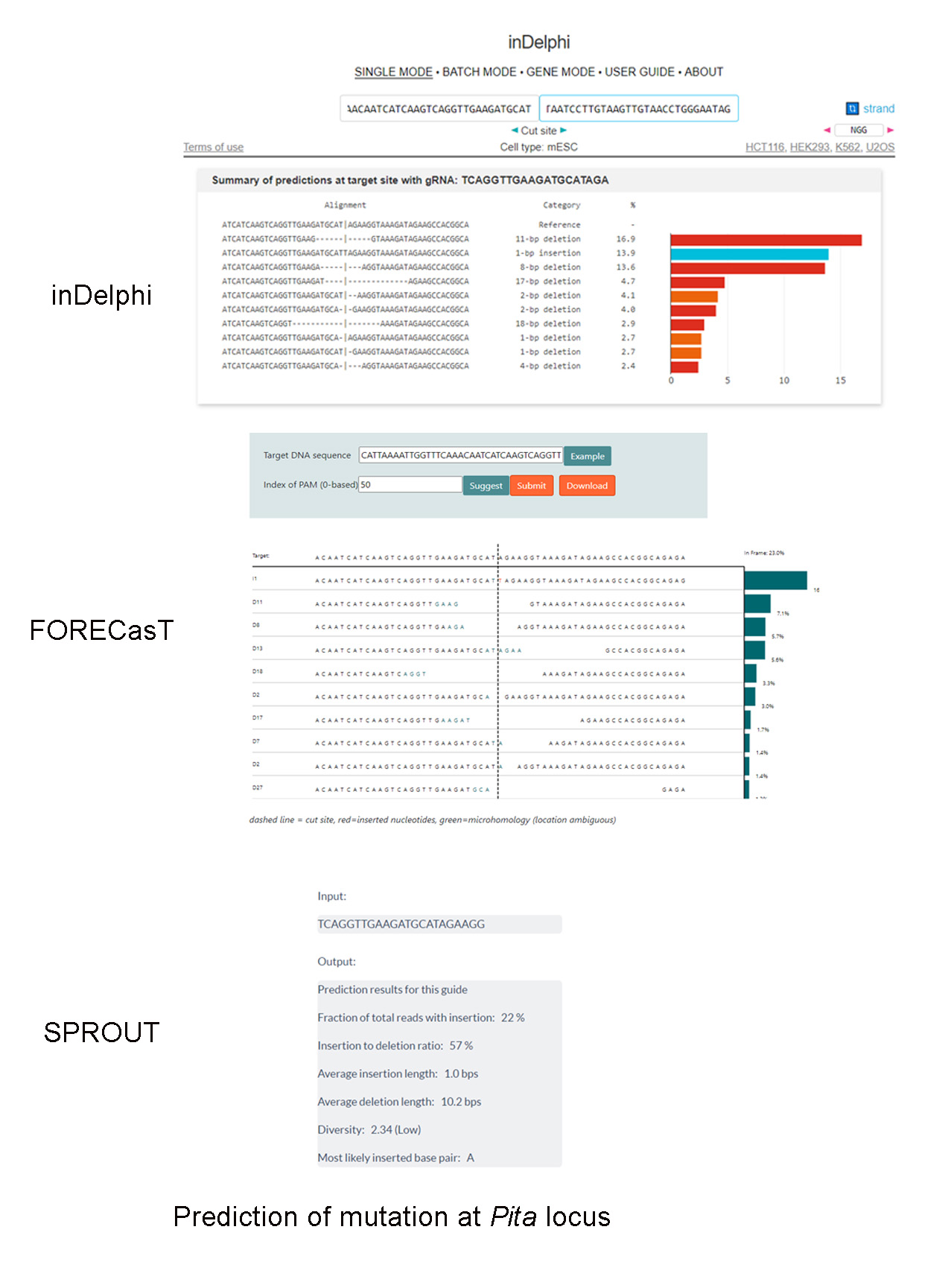


**Supplementary Figure 5:** Representative screenshot showing prediction of Cas9 repair outcome at the *Pita* locus.


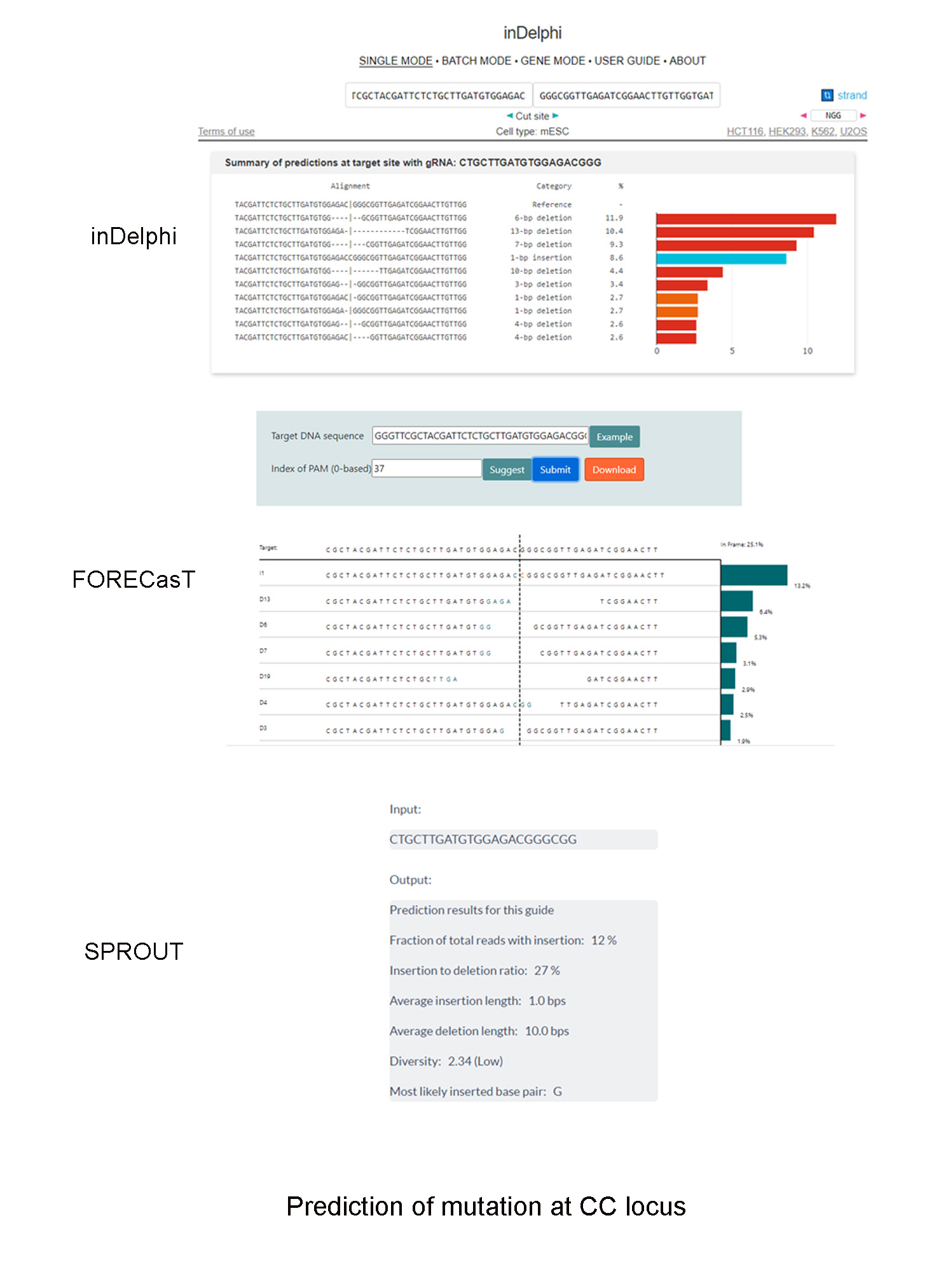


**Supplementary Figure 6:** Representative screenshot showing prediction of Cas9 repair outcome at the *CC* locus.


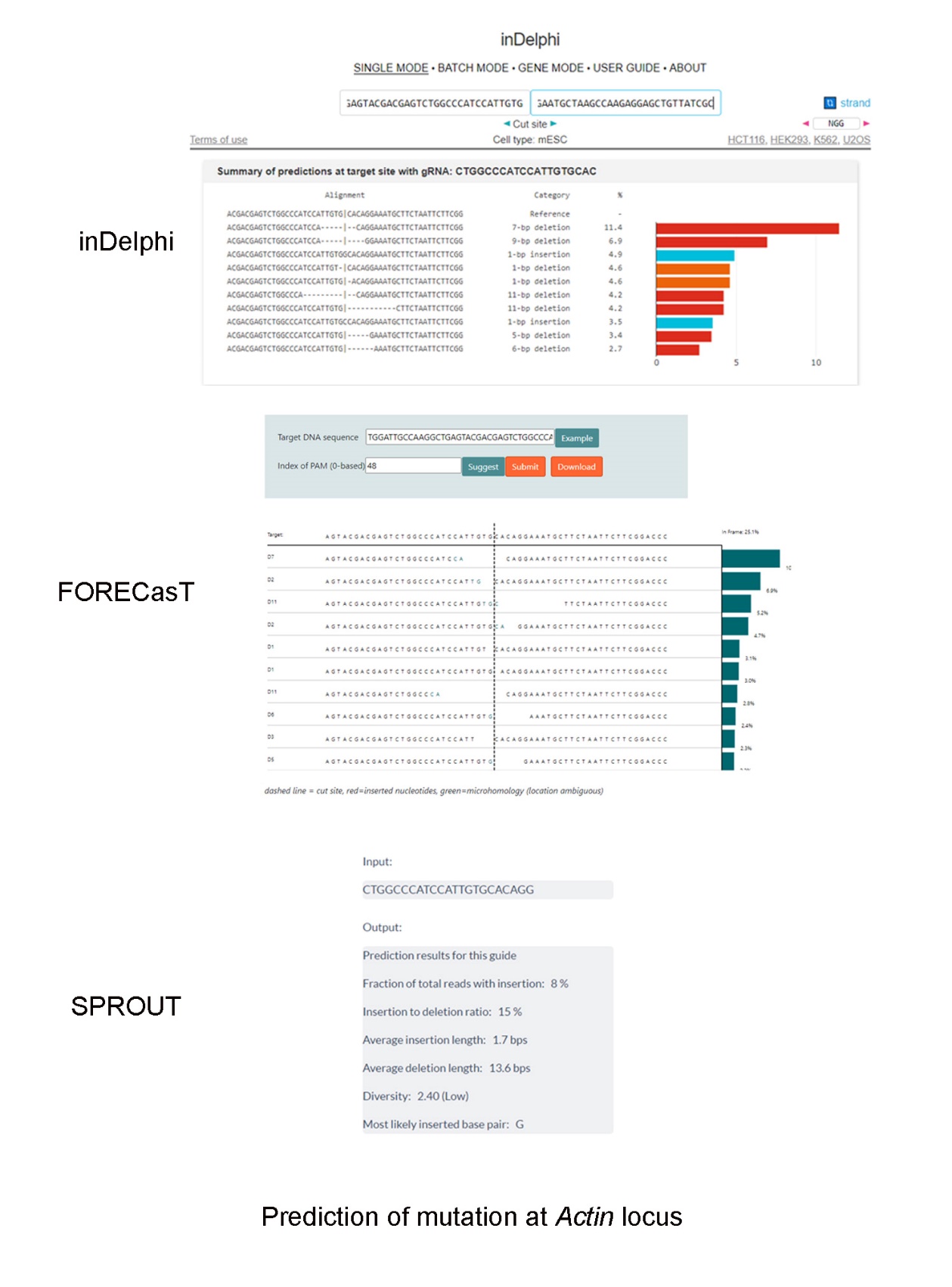


**Supplementary Figure 7:** Representative screenshot showing prediction of Cas9 repair outcome at the *Actin* locus.

**Supplementary Sequences:** Annotated nucleotide sequence for constructs used in this study

**CRISPEY (GFP to BFP) in *N. benthamiana*** (sequences within T-DNA border is shown. Selectable marker cassette is not shown)

| **Colour/other code (sequential)** | **Sequence detail** |
| --- | --- |
| Yellow: | AtU6 promoter |
| Green: | tRNA |
| **Bold:** | Retron including donor seq |
| Underlined: | msr sequence |
| Turquoise: | donor template (intended changes are shown in capital letter) |
| Pink: | mGFP protospacer |
| Gray: | SpCas9 scaffold |
| Teal: | HDV ribozyme |
| Red font | CaMV35S promoter |
| Green font | SV40 NLS |
| Purple font | Codon optimized EcRT86 |
| Orange font | Self-cleaving peptide P2A |
| Blue font | SpCas9 |
| Teal font | Nucleoplasmin NLS |
| Gold font | Nos terminator |

tgatcaaaagtcccacatcgatcaggtgatatatagcagcttagtttatataatgatagagtcgacatagcgattGgagaccaacaaagcaccagtggtctagtggtagaatagtaccctgccacggtacagacccgggttcgattcccggctggtgca**ATGCGCACCCTTAGCGAGAGGTTTATCATTAAGGTCAACCTCTGGATGTTGTTTCGGCATCCTGCATTGAATCTGAGTTACTGTCTGTTTTCCTaacttacccttaaatttatttgcactactggaaaactacctgttccatggccaacacttgtcactactttTAGtCatggtgttcaatgcttttcaagatacccagatcatatgaagcggcacgacttcttcaagagcgccatgccAGGAAACCCGTTTCTTCTGACGTAAGGGTGCGCA**cttgtcactactttctcttagttttagagctagaaatagcaagttaaaataaggctagtccgttatcaacttgaaaaagtggcaccgagtcggtgcGATGGCCGGCATGGTCCCAGCCTCCTCGCTGGCGCCGGCTGGGCAACACCTTCGGGTGGCGAATGGGACTTtttttttggtctcttttCTCAAcctgcaggtccatgagacttttcaacaaaggataatttcgggaaacctcctcggattccattgcccagctatctgtcacttcatcgaaaggacagtagaaaaggaaggtggctcctacaaatgccatcattgcgataaaggaaaggctatcattcaagatctctctgccgacagtggtcccaaagatggacccccacccacgaggagcatcgtggaaaaagaagaggttccaaccacgtctacaaagcaagtggattgatgtgacatctccactgacgtaagggatgacgcacaatcccactatccttcgcaagacccttcctctatataaggaagttcatttcatttggagaggacaGGATCCCCGCCATGCCGCCTAAAAAAAAAAGAAAGGTCAAGTCCGCCGAGTATTTAAACACCTTTCGACTCAGGAATCTGGGTTTGCCTGTCATGAACAATCTTCACGATATGTCCAAGGCAACGCGTATTTCTGTGGAAACTCTGCGACTGTTAATCTACACGGCTGACTTCCGATACCGAATTTACACAGTTGAAAAAAAGGGTCCTGAGAAGAGGATGAGAACGATATACCAGCCGAGTAGGGAACTGAAGGCTTTACAAGGTTGGGTATTACGTAATATCTTAGACAAGCTGTCCTCCTCTCCATTCTCAATAGGTTTTGAGAAGCATCAGTCAATACTTAACAACGCTACTCCTCACATCGGTGCTAATTTTATCCTTAACATAGACTTAGAGGACTTTTTTCCGAGTTTAACAGCCAATAAAGTTTTCGGGGTTTTCCATAGCCTGGGGTATAACCGATTGATATCTAGTGTTTTAACTAAAATATGTTGCTACAAAAACCTGCTCCCTCAAGGGGCTCCCAGCTCTCCAAAGCTGGCAAACCTCATATGTAGTAAGCTGGACTATCGAATTCAAGGGTACGCAGGCTCTAGAGGGCTCATTTATACTCGTTATGCTGACGATTTGACGCTCTCTGCACAATCTATGAAAAAGGTTGTAAAAGCCCGTGACTTTCTTTTTAGTATTATTCCTTCAGAGGGTTTAGTAATCAATTCCAAGAAGACCTGCATCTCTGGGCCGCGAAGTCAACGAAAGGTAACGGGTTTGGTCATTAGTCAAGAAAAGGTTGGTATAGGACGTGAAAAATATAAAGAGATTCGAGCAAAGATTCATCACATTTTTTGTGGTAAAAGTAGCGAAATCGAACATGTTAGAGGCTGGCTTTCATTCATCCTCTCCGTGGACTCAAAGTCCCATCGTCGATTAATAACGTACATCTCAAAACTGGAAAAGAAATACGGGAAAAATCCATTGAACAAGGCCAAAACTGGCTCCGGAGCTACGAACTTTAGCTTGTTAAAGCAGGCTGGTGATGTGGAGGAAAACCCAGGACCTcccatggactataaggaccacgacggagactacaaggatcatgatattgattacaaagacgatgacgataagatggccCCAAAGAAGAAGCGGAAGGTCggtatccacggagtcccagcagccgacaagaagtacagcatcggcctggacatcggcaccaactctgtgggctgggccgtgatcaccgacgagtacaaggtgcccagcaagaaattcaaggtgctgggcaacaccgaccggcacagcatcaagaagaacctgatcggagccctgctgttcgacagcggcgaaacagccgaggccacccggctgaagagaaccgccagaagaagatacaccagacggaagaaccggatctgctatctgcaagagatcttcagcaacgagatggccaaggtggacgacagcttcttccacagactggaagagtccttcctggtggaagaggataagaagcacgagcggcaccccatcttcggcaacatcgtggacgaggtggcctaccacgagaagtaccccaccatctaccacctgagaaagaaactggtggacagcaccgacaaggccgacctgcggctgatctatctggccctggcccacatgatcaagttccggggccacttcctgatcgagggcgacctgaaccccgacaacagcgacgtggacaagctgttcatccagctggtgcagacctacaaccagctgttcgaggaaaaccccatcaacgccagcggcgtggacgccaaggccatcctgtctgccagactgagcaagagcagacggctggaaaatctgatcgcccagctgcccggcgagaagaagaatggcctgttcggaaacctgattgccctgagcctgggcctgacccccaacttcaagagcaacttcgacctggccgaggatgccaaactgcagctgagcaaggacacctacgacgacgacctggacaacctgctggcccagatcggcgaccagtacgccgacctgtttctggccgccaagaacctgtccgacgccatcctgctgagcgacatcctgagagtgaacaccgagatcaccaaggcccccctgagcgcctctatgatcaagagatacgacgagcaccaccaggacctgaccctgctgaaagctctcgtgcggcagcagctgcctgagaagtacaaagagattttcttcgaccagagcaagaacggctacgccggctacattgacggcggagccagccaggaagagttctacaagttcatcaagcccatcctggaaaagatggacggcaccgaggaactgctcgtgaagctgaacagagaggacctgctgcggaagcagcggaccttcgacaacggcagcatcccccaccagatccacctgggagagctgcacgccattctgcggcggcaggaagatttttacccattcctgaaggacaaccgggaaaagatcgagaagatcctgaccttccgcatcccctactacgtgggccctctggccaggggaaacagcagattcgcctggatgaccagaaagagcgaggaaaccatcaccccctggaacttcgaggaagtggtggacaagggcgcttccgcccagagcttcatcgagcggatgaccaacttcgataagaacctgcccaacgagaaggtgctgcccaagcacagcctgctgtacgagtacttcaccgtgtataacgagctgaccaaagtgaaatacgtgaccgagggaatgagaaagcccgccttcctgagcggcgagcagaaaaaggccatcgtggacctgctgttcaagaccaaccggaaagtgaccgtgaagcagctgaaagaggactacttcaagaaaatcgagtgcttcgactccgtggaaatctccggcgtggaagatcggttcaacgcctccctgggcacataccacgatctgctgaaaattatcaaggacaaggacttcctggacaatgaggaaaacgaggacattctggaagatatcgtgctgaccctgacactgtttgaggacagagagatgatcgaggaacggctgaaaacctatgcccacctgttcgacgacaaagtgatgaagcagctgaagcggcggagatacaccggctggggcaggctgagccggaagctgatcaacggcatccgggacaagcagtccggcaagacaatcctggatttcctgaagtccgacggcttcgccaacagaaacttcatgcagctgatccacgacgacagcctgacctttaaagaggacatccagaaagcccaggtgtccggccagggcgatagcctgcacgagcacattgccaatctggccggcagccccgccattaagaagggcatcctgcagacagtgaaggtggtggacgagctcgtgaaagtgatgggccggcacaagcccgagaacatcgtgatcgaaatggccagagagaaccagaccacccagaagggacagaagaacagccgcgagagaatgaagcggatcgaagagggcatcaaagagctgggcagccagatcctgaaagaacaccccgtggaaaacacccagctgcagaacgagaagctgtacctgtactacctgcagaatgggcgggatatgtacgtggaccaggaactggacatcaaccggctgtccgactacgatgtggaccatatcgtgcctcagagctttctgaaggacgactccatcgacaacaaggtgctgaccagaagcgacaagaaccggggcaagagcgacaacgtgccctccgaagaggtcgtgaagaagatgaagaactactggcggcagctgctgaacgccaagctgattacccagagaaagttcgacaatctgaccaaggccgagagaggcggcctgagcgaactggataaggccggcttcatcaagagacagctggtggaaacccggcagatcacaaagcacgtggcacagatcctggactcccggatgaacactaagtacgacgagaatgacaagctgatccgggaagtgaaagtgatcaccctgaagtccaagctggtgtccgatttccggaaggatttccagttttacaaagtgcgcgagatcaacaactaccaccacgcccacgacgcctacctgaacgccgtcgtgggaaccgccctgatcaaaaagtaccctaagctggaaagcgagttcgtgtacggcgactacaaggtgtacgacgtgcggaagatgatcgccaagagcgagcaggaaatcggcaaggctaccgccaagtacttcttctacagcaacatcatgaactttttcaagaccgagattaccctggccaacggcgagatccggaagcggcctctgatcgagacaaacggcgaaaccggggagatcgtgtgggataagggccgggattttgccaccgtgcggaaagtgctgagcatgccccaagtgaatatcgtgaaaaagaccgaggtgcagacaggcggcttcagcaaagagtctatcctgcccaagaggaacagcgataagctgatcgccagaaagaaggactgggaccctaagaagtacggcggcttcgacagccccaccgtggcctattctgtgctggtggtggccaaagtggaaaagggcaagtccaagaaactgaagagtgtgaaagagctgctggggatcaccatcatggaaagaagcagcttcgagaagaatcccatcgactttctggaagccaagggctacaaagaagtgaaaaaggacctgatcatcaagctgcctaagtactccctgttcgagctggaaaacggccggaagagaatgctggcctctgccggcgaactgcagaagggaaacgaactggccctgccctccaaatatgtgaacttcctgtacctggccagccactatgagaagctgaagggctcccccgaggataatgagcagaaacagctgtttgtggaacagcacaagcactacctggacgagatcatcgagcagatcagcgagttctccaagagagtgatcctggccgacgctaatctggacaaagtgctgtccgcctacaacaagcaccgggataagcccatcagagagcaggccgagaatatcatccacctgtttaccctgaccaatctgggagcccctgccgccttcaagtactttgacaccaccatcgaccggaagaggtacaccagcaccaaagaggtgctggacgccaccctgatccaccagagcatcaccggcctgtacgagacacggatcgacctgtctcagctgggaggcgacAAAAGGCCGGCGGCCACGAAAAAGGCCGGCCAGGCAAAAAAGAAAAAGtaagaattcgcggccgcactcgagatatctagacccagctttcttgtacaaagtggttgataacagcgactacaaggatgacgatgacaaggcttagagctcgaatttccccgatcgttcaaacatttggcaataaagtttcttaagattgaatcctgttgccggtcttgcgatgattatcatataatttctgttgaattacgttaagcatgtaataattaacatgtaatgcatgacgttatttatgagatgggtttttatgattagagtcccgcaattatacatttaatacgcgatagaaaacaaaatatagcgcgcaaactaggataaattatcgcgcgcggtgtcatctatgttactagatcgggaat

**pK-CRISPEY-ALS** (sequences within T-DNA border is shown. Selectable marker cassette is not shown)

| **Colour/other code (sequential)** | **Sequence detail** |
| --- | --- |
| Magenta font | OsUbi10 promoter |
| Green font | SV40 NLS |
| Purple font | Codon optimized EcRT86 |
| Cyan | Gene 7 terminator |
| Red font | CaMV35S promoter (enhanced) |
| Dark Yellow | HH ribozyme |
| **Bold:** | Retron including donor seq |
| Underlined: | msr sequence |
| Turquoise: | donor template (intended changes are shown in small letter) |
| Pink: | ALS protospacer |
| Gray: | SpCas9 scaffold |
| Teal: | HDV ribozyme |
| Red: | HSP terminator |
| Blue font | SpCas9 |
| Teal font | Nucleoplasmin NLS |
| Gold font | Nos terminator |

attcgggtcaaggcggaagccagcgcgccaccccacgtcagcaaatacggaggcgcggggttgacggcgtcacccggtcctaacggcgaccaacaaaccagccagaagaaattacagtaaaaaaaaagtaaattgcactttgatccaccttttattacctaagtctcaatttggatcacccttaaacctatcttttcaatttgggccgggttgtggtttggactaccatgaacaacttttcgtcatgtctaacttccctttcagcaaacatatgaaccatatatagaggagatcggccgtatactagagctgatgtgtttaaggtcgttgattgcacgagaaaaaaaaatccaaatcgcaacaatagcaaatttatctggttcaaagtgaaaagatatgtttaaaggtagtccaaagtaaaacttatagataataaaatgtggtccaaagcgtaattcactcaaaaaaaatcaacgagacgtgtaccaaacggagacaaacggcatcttctcgaaatttcccaaccgctcgctcgcccgcctcgtcttcccggaaaccgcggtggtttcagcgtggcggattctccaagcagacggagacgtcacggcacgggactcctcccaccacccaaccgccataaataccagccccctcatctcctctcctcgcatcagctccacccccgaaaaatttctccccaatctcgcgaggctctcgtcgtcgaatcgaatcctctcgcgtcctcaaggtacgctgcttctcctctcctcgcttcgtttcgattcgatttcggacgggtgaggttgttttgttgctagatccgattggtggttagggttgtcgatgtgattatcgtgagatgtttaggggttgtagatctgatggttgtgatttgggcacggttggttcgataggtggaatcgtggttaggttttgggattggatgttggttctgatgattggggggaatttttacggttagatgaattgttggatgattcgattggggaaatcggtgtagatctgttggggaattgtggaactagtcatgcctgagtgattggtgcgatttgtagcgtgttccatcttgtaggccttgttgcgagcatgttcagatctactgttccgctcttgattgagttattggtgccatgggttggtgcaaacacaggctttaatatgttatatctgttttgtgtttgatgtagatctgtagggtagttcttcttagacatggttcaattatgtagcttgtgcgtttcgatttgatttcatatgttcacagattagataatgatgaactcttttaattaattgtcaatggtaaataggaagtcttgtcgctatatctgtcataatgatctcatgttactatctgccagtaatttatgctaagaactatattagaatatcatgttacaatctgtagtaatatcatgttacaatctgtagttcatctatataatctattgtggtaatttctttttactatctgtgtgaagattattgccactagttcattctacttatttctgaagttcaggatacgtgtgctgttactacctatctgaatacatgtgtgatgtgcctgttactatctttttgaatacatgtatgttctgttggaatatgtttgctgtttgatccgttgttgtgtccttaatcttgtgctagttcttaccctatctgtttggtgattatttcttgcagatagttatcaacaagtttgtacCGAGCTCGGATCCACACCATGGCGCCTAAGAAGAAGAGAAAAGTTAAAAGTGCCGAGTACCTCAATACGTTTAGACTGAGGAACTTGGGGCTTCCAGTGATGAATAACTTGCATGACATGTCAAAGGCGACGCGCATTTCAGTTGAAACTCTCAGGTTGCTCATCTATACGGCCGACTTTAGGTACCGGATCTACACGGTTGAGAAGAAGGGCCCGGAGAAACGGATGAGGACCATTTACCAACCTTCGAGAGAGCTGAAAGCCCTCCAGGGTTGGGTCCTGAGGAACATTCTGGATAAACTTTCTTCATCCCCCTTCAGCATCGGGTTCGAGAAACATCAGTCGATACTGAATAATGCAACTCCTCACATCGGAGCGAACTTTATCCTCAACATCGACTTGGAAGATTTCTTTCCGTCTTTGACAGCTAATAAAGTGTTTGGGGTGTTTCACAGTCTTGGTTATAACAGGCTTATTTCATCAGTTCTCACCAAAATCTGCTGTTATAAGAATCTTCTTCCACAGGGGGCACCAAGCTCACCAAAGTTGGCGAACCTGATTTGTTCCAAGCTCGACTACAGGATACAGGGGTATGCAGGAAGTCGCGGACTTATCTACACAAGGTATGCGGACGACCTGACTCTCTCTGCACAATCCATGAAGAAAGTTGTGAAGGCCAGGGACTTCTTGTTCTCTATCATTCCTTCTGAGGGCCTCGTTATCAACAGTAAGAAAACTTGCATCAGCGGGCCGAGGTCACAGAGAAAGGTGACAGGGTTGGTTATCAGTCAAGAGAAAGTGGGAATTGGTAGAGAGAAATACAAGGAAATACGCGCAAAGATCCACCATATATTCTGTGGTAAAAGTAGCGAGATCGAACACGTTAGAGGCTGGCTGTCGTTTATCTTGTCTGTCGACTCAAAGTCCCATAGGCGCCTTATCACGTATATCAGTAAACTTGAAAAGAAATACGGAAAGAATCCACTGAACAAAGCTAAAACTTGAcccgatgagctaaGGATCCCCCGGGgctagctatatcatcaatttatgtattacacataatatcgcactcagtctttcatctacggcaatgtaccagctgatataatcagttattgaaatatttctgaatttaaacttgcatcaataaatttatgtttttgcttggactataatacctgacttgttattttatcaataaatatttaaactatatttctttcaagatTTTTTTGGTGGAGCACGACACTCTCGTCTACTCCAAGAATATCAAAGATACAGTCTCAGAAGACCAAAGGGCTATTGAGACTTTTCAACAAAGGGTAATATCGGGAAACCTCCTCGGATTCCATTGCCCAGCTATCTGTCACTTCATCAAAAGGACAGTAGAAAAGGAAGGTGGCACCTACAAATGCCATCATTGCGATAAAGGAAAGGCTATCGTTCAAGATGCCTCTGCCGACAGTGGTCCCAAAGATGGACCCCCACCCACGAGGAGCATCGTGGAAAAAGAAGACGTTCCAACCACGTCTTCAAAGCAAGTGGATTGATGTGATAACATGGTGGAGCACGACACTCTCGTCTACTCCAAGAATATCAAAGATACAGTCTCAGAAGACCAAAGGGCTATTGAGACTTTTCAACAAAGGGTAATATCGGGAAACCTCCTCGGATTCCATTGCCCAGCTATCTGTCACTTCATCAAAAGGACAGTAGAAAAGGAAGGTGGCACCTACAAATGCCATCATTGCGATAAAGGAAAGGCTATCGTTCAAGATGCCTCTGCCGACAGTGGTCCCAAAGATGGACCCCCACCCACGAGGAGCATCGTGGAAAAAGAAGACGTTCCAACCACGTCTTCAAAGCAAGTGGATTGATGTGATATCTCCACTGACGTAAGGGATGACGCACAATCCCACTATCCTTCGCAAGACCTTCCTCTATATAAGGAAGTTCATTTCATTTGGAGAGGACACGCTGAAATCACCAGTCTCTCTCTACAAATCTATCTCTGGGTGCGCATctgatgagtccgtgaggacgaaacgagctagctcgtc**ATGCGCACCCTTAGCGAGAGGTTTATCATTAAGGTCAACCTCTGGATGTTGTTTCGGCATCCTGCATTGAATCTGAGTTACTGTCTGTTTTCCTGGTGAAGGTGATGGTGTTGAACAACCAACATTTGGGTATGGTTGTGCAATtGGAaGATAGGTTTTACAAGGCAAATAGGGCGCATACATACTTGGGCAACAGGAAACCCGTTTCTTCTGACGTAAGGGTGCGCA**GGGTATGGTTGTGCAATGGGgttttagagctagaaatagcaagttaaaataaggctagtccgttatcaacttgaaaaagtggcaccgagtcggtgcGATGGCCGGCATGGTCCCAGCCTCCTCGCTGGCGCCGGCTGGGCAACACCTTCGGGTGGCGAATGGGACTTTATGAAGATGAAGATGAAATATTTGGTGTGTCAAATAAAAAGCTAGCTTGTGTGCTTAAGTTTGTGTTTTTTTCTTGGCTTGTTGTGTTATGAATTTGTGGCTTTTTCTAATATTAAATGAATGTAAGATCTCATTATAATGAATAAACAAATGTTTCTATAATCCATTGTGAATGTTTTGTTGGATCTCTTCGCATATAACTACTGTATGTGCTATGGTATGGACTATGGAATATGATTAAAGATAAGaggtctcggttttagagctagaaatagcaagttaaaataaggctagtccgttatcaacttgaaaaagtggcaccgagtcggtgcttttttgttttagagctagaaatagcaagttaaaataaggctagtccgtttttagcgcgtgcatgcctgcaggtccacaaattcgggtcaaggcggaagccagcgcgccaccccacgtcagcaaatacggaggcgcggggttgacggcgtcacccggtcctaacggcgaccaacaaaccagccagaagaaattacagtaaaaaaaaagtaaattgcactttgatccaccttttattacctaagtctcaatttggatcacccttaaacctatcttttcaatttgggccgggttgtggtttggactaccatgaacaacttttcgtcatgtctaacttccctttcagcaaacatatgaaccatatatagaggagatcggccgtatactagagctgatgtgtttaaggtcgttgattgcacgagaaaaaaaaatccaaatcgcaacaatagcaaatttatctggttcaaagtgaaaagatatgtttaaaggtagtccaaagtaaaacttatagataataaaatgtggtccaaagcgtaattcactcaaaaaaaatcaacgagacgtgtaccaaacggagacaaacggcatcttctcgaaatttcccaaccgctcgctcgcccgcctcgtcttcccggaaaccgcggtggtttcagcgtggcggattctccaagcagacggagacgtcacggcacgggactcctcccaccacccaaccgccataaataccagccccctcatctcctctcctcgcatcagctccacccccgaaaaatttctccccaatctcgcgaggctctcgtcgtcgaatcgaatcctctcgcgtcctcaaggtacgctgcttctcctctcctcgcttcgtttcgattcgatttcggacgggtgaggttgttttgttgctagatccgattggtggttagggttgtcgatgtgattatcgtgagatgtttaggggttgtagatctgatggttgtgatttgggcacggttggttcgataggtggaatcgtggttaggttttgggattggatgttggttctgatgattggggggaatttttacggttagatgaattgttggatgattcgattggggaaatcggtgtagatctgttggggaattgtggaactagtcatgcctgagtgattggtgcgatttgtagcgtgttccatcttgtaggccttgttgcgagcatgttcagatctactgttccgctcttgattgagttattggtgccatgggttggtgcaaacacaggctttaatatgttatatctgttttgtgtttgatgtagatctgtagggtagttcttcttagacatggttcaattatgtagcttgtgcgtttcgatttgatttcatatgttcacagattagataatgatgaactcttttaattaattgtcaatggtaaataggaagtcttgtcgctatatctgtcataatgatctcatgttactatctgccagtaatttatgctaagaactatattagaatatcatgttacaatctgtagtaatatcatgttacaatctgtagttcatctatataatctattgtggtaatttctttttactatctgtgtgaagattattgccactagttcattctacttatttctgaagttcaggatacgtgtgctgttactacctatctgaatacatgtgtgatgtgcctgttactatctttttgaatacatgtatgttctgttggaatatgtttgctgtttgatccgttgttgtgtccttaatcttgtgctagttcttaccctatctgtttggtgattatttcttgcagatagttatcaacaagtttgtacaaaaaagcaggcttcgaaggagatagaaccaattctctaaggaaatacttaaccatggactataaggaccacgacggagactacaaggatcatgatattgattacaaagacgatgacgataagatggccCCAAAGAAGAAGCGGAAGGTCggtatccacggagtcccagcagccgacaagaagtacagcatcggcctggacatcggcaccaactctgtgggctgggccgtgatcaccgacgagtacaaggtgcccagcaagaaattcaaggtgctgggcaacaccgaccggcacagcatcaagaagaacctgatcggagccctgctgttcgacagcggcgaaacagccgaggccacccggctgaagagaaccgccagaagaagatacaccagacggaagaaccggatctgctatctgcaagagatcttcagcaacgagatggccaaggtggacgacagcttcttccacagactggaagagtccttcctggtggaagaggataagaagcacgagcggcaccccatcttcggcaacatcgtggacgaggtggcctaccacgagaagtaccccaccatctaccacctgagaaagaaactggtggacagcaccgacaaggccgacctgcggctgatctatctggccctggcccacatgatcaagttccggggccacttcctgatcgagggcgacctgaaccccgacaacagcgacgtggacaagctgttcatccagctggtgcagacctacaaccagctgttcgaggaaaaccccatcaacgccagcggcgtggacgccaaggccatcctgtctgccagactgagcaagagcagacggctggaaaatctgatcgcccagctgcccggcgagaagaagaatggcctgttcggaaacctgattgccctgagcctgggcctgacccccaacttcaagagcaacttcgacctggccgaggatgccaaactgcagctgagcaaggacacctacgacgacgacctggacaacctgctggcccagatcggcgaccagtacgccgacctgtttctggccgccaagaacctgtccgacgccatcctgctgagcgacatcctgagagtgaacaccgagatcaccaaggcccccctgagcgcctctatgatcaagagatacgacgagcaccaccaggacctgaccctgctgaaagctctcgtgcggcagcagctgcctgagaagtacaaagagattttcttcgaccagagcaagaacggctacgccggctacattgacggcggagccagccaggaagagttctacaagttcatcaagcccatcctggaaaagatggacggcaccgaggaactgctcgtgaagctgaacagagaggacctgctgcggaagcagcggaccttcgacaacggcagcatcccccaccagatccacctgggagagctgcacgccattctgcggcggcaggaagatttttacccattcctgaaggacaaccgggaaaagatcgagaagatcctgaccttccgcatcccctactacgtgggccctctggccaggggaaacagcagattcgcctggatgaccagaaagagcgaggaaaccatcaccccctggaacttcgaggaagtggtggacaagggcgcttccgcccagagcttcatcgagcggatgaccaacttcgataagaacctgcccaacgagaaggtgctgcccaagcacagcctgctgtacgagtacttcaccgtgtataacgagctgaccaaagtgaaatacgtgaccgagggaatgagaaagcccgccttcctgagcggcgagcagaaaaaggccatcgtggacctgctgttcaagaccaaccggaaagtgaccgtgaagcagctgaaagaggactacttcaagaaaatcgagtgcttcgactccgtggaaatctccggcgtggaagatcggttcaacgcctccctgggcacataccacgatctgctgaaaattatcaaggacaaggacttcctggacaatgaggaaaacgaggacattctggaagatatcgtgctgaccctgacactgtttgaggacagagagatgatcgaggaacggctgaaaacctatgcccacctgttcgacgacaaagtgatgaagcagctgaagcggcggagatacaccggctggggcaggctgagccggaagctgatcaacggcatccgggacaagcagtccggcaagacaatcctggatttcctgaagtccgacggcttcgccaacagaaacttcatgcagctgatccacgacgacagcctgacctttaaagaggacatccagaaagcccaggtgtccggccagggcgatagcctgcacgagcacattgccaatctggccggcagccccgccattaagaagggcatcctgcagacagtgaaggtggtggacgagctcgtgaaagtgatgggccggcacaagcccgagaacatcgtgatcgaaatggccagagagaaccagaccacccagaagggacagaagaacagccgcgagagaatgaagcggatcgaagagggcatcaaagagctgggcagccagatcctgaaagaacaccccgtggaaaacacccagctgcagaacgagaagctgtacctgtactacctgcagaatgggcgggatatgtacgtggaccaggaactggacatcaaccggctgtccgactacgatgtggaccatatcgtgcctcagagctttctgaaggacgactccatcgacaacaaggtgctgaccagaagcgacaagaaccggggcaagagcgacaacgtgccctccgaagaggtcgtgaagaagatgaagaactactggcggcagctgctgaacgccaagctgattacccagagaaagttcgacaatctgaccaaggccgagagaggcggcctgagcgaactggataaggccggcttcatcaagagacagctggtggaaacccggcagatcacaaagcacgtggcacagatcctggactcccggatgaacactaagtacgacgagaatgacaagctgatccgggaagtgaaagtgatcaccctgaagtccaagctggtgtccgatttccggaaggatttccagttttacaaagtgcgcgagatcaacaactaccaccacgcccacgacgcctacctgaacgccgtcgtgggaaccgccctgatcaaaaagtaccctaagctggaaagcgagttcgtgtacggcgactacaaggtgtacgacgtgcggaagatgatcgccaagagcgagcaggaaatcggcaaggctaccgccaagtacttcttctacagcaacatcatgaactttttcaagaccgagattaccctggccaacggcgagatccggaagcggcctctgatcgagacaaacggcgaaaccggggagatcgtgtgggataagggccgggattttgccaccgtgcggaaagtgctgagcatgccccaagtgaatatcgtgaaaaagaccgaggtgcagacaggcggcttcagcaaagagtctatcctgcccaagaggaacagcgataagctgatcgccagaaagaaggactgggaccctaagaagtacggcggcttcgacagccccaccgtggcctattctgtgctggtggtggccaaagtggaaaagggcaagtccaagaaactgaagagtgtgaaagagctgctggggatcaccatcatggaaagaagcagcttcgagaagaatcccatcgactttctggaagccaagggctacaaagaagtgaaaaaggacctgatcatcaagctgcctaagtactccctgttcgagctggaaaacggccggaagagaatgctggcctctgccggcgaactgcagaagggaaacgaactggccctgccctccaaatatgtgaacttcctgtacctggccagccactatgagaagctgaagggctcccccgaggataatgagcagaaacagctgtttgtggaacagcacaagcactacctggacgagatcatcgagcagatcagcgagttctccaagagagtgatcctggccgacgctaatctggacaaagtgctgtccgcctacaacaagcaccgggataagcccatcagagagcaggccgagaatatcatccacctgtttaccctgaccaatctgggagcccctgccgccttcaagtactttgacaccaccatcgaccggaagaggtacaccagcaccaaagaggtgctggacgccaccctgatccaccagagcatcaccggcctgtacgagacacggatcgacctgtctcagctgggaggcgacAAAAGGCCGGCGGCCACGAAAAAGGCCGGCCAGGCAAAAAAGAAAAAGtaagaattcgcggccgcactcgagatatctagacccagctttcttgtacaaagtggttgataacagcgactacaaggatgacgatgacaaggcttagagctcgaatttccccgatcgttcaaacatttggcaataaagtttcttaagattgaatcctgttgccggtcttgcgatgattatcatataatttctgttgaattacgttaagcatgtaataattaacatgtaatgcatgacgttatttatgagatgggtttttatgattagagtcccgcaattatacatttaatacgcgatagaaaacaaaatatagcgcgcaaactaggataaattatcgcgcgcggtgtcatctatgttactagatcgggaa

**pK-CRISPEY-CC** (sequences within T-DNA border is shown. Selectable marker cassette is not shown)

| **Colour/other code (sequential)** | **Sequence detail** |
| --- | --- |
| Magenta font | OsUbi10 promoter |
| Green font | SV40 NLS |
| Purple font | Codon optimized EcRT86 |
| Brown | Gene 7 terminator |
| Red font | CaMV35S promoter (enhanced) |
| Dark Yellow | HH ribozyme |
| **Bold:** | Retron including donor seq |
| Underlined: | msr sequence |
| Turquoise: | donor template (intended changes are shown in small letter) |
| Pink: | CC protospacer |
| Gray: | SpCas9 scaffold |
| Teal: | HDV ribozyme |
| Red: | HSP terminator |
| Blue font | SpCas9 |
| Teal font | Nucleoplasmin NLS |
| Gold font | Nos terminator |

attcgggtcaaggcggaagccagcgcgccaccccacgtcagcaaatacggaggcgcggggttgacggcgtcacccggtcctaacggcgaccaacaaaccagccagaagaaattacagtaaaaaaaaagtaaattgcactttgatccaccttttattacctaagtctcaatttggatcacccttaaacctatcttttcaatttgggccgggttgtggtttggactaccatgaacaacttttcgtcatgtctaacttccctttcagcaaacatatgaaccatatatagaggagatcggccgtatactagagctgatgtgtttaaggtcgttgattgcacgagaaaaaaaaatccaaatcgcaacaatagcaaatttatctggttcaaagtgaaaagatatgtttaaaggtagtccaaagtaaaacttatagataataaaatgtggtccaaagcgtaattcactcaaaaaaaatcaacgagacgtgtaccaaacggagacaaacggcatcttctcgaaatttcccaaccgctcgctcgcccgcctcgtcttcccggaaaccgcggtggtttcagcgtggcggattctccaagcagacggagacgtcacggcacgggactcctcccaccacccaaccgccataaataccagccccctcatctcctctcctcgcatcagctccacccccgaaaaatttctccccaatctcgcgaggctctcgtcgtcgaatcgaatcctctcgcgtcctcaaggtacgctgcttctcctctcctcgcttcgtttcgattcgatttcggacgggtgaggttgttttgttgctagatccgattggtggttagggttgtcgatgtgattatcgtgagatgtttaggggttgtagatctgatggttgtgatttgggcacggttggttcgataggtggaatcgtggttaggttttgggattggatgttggttctgatgattggggggaatttttacggttagatgaattgttggatgattcgattggggaaatcggtgtagatctgttggggaattgtggaactagtcatgcctgagtgattggtgcgatttgtagcgtgttccatcttgtaggccttgttgcgagcatgttcagatctactgttccgctcttgattgagttattggtgccatgggttggtgcaaacacaggctttaatatgttatatctgttttgtgtttgatgtagatctgtagggtagttcttcttagacatggttcaattatgtagcttgtgcgtttcgatttgatttcatatgttcacagattagataatgatgaactcttttaattaattgtcaatggtaaataggaagtcttgtcgctatatctgtcataatgatctcatgttactatctgccagtaatttatgctaagaactatattagaatatcatgttacaatctgtagtaatatcatgttacaatctgtagttcatctatataatctattgtggtaatttctttttactatctgtgtgaagattattgccactagttcattctacttatttctgaagttcaggatacgtgtgctgttactacctatctgaatacatgtgtgatgtgcctgttactatctttttgaatacatgtatgttctgttggaatatgtttgctgtttgatccgttgttgtgtccttaatcttgtgctagttcttaccctatctgtttggtgattatttcttgcagatagttatcaacaagtttgtacCGAGCTCGGATCCACACCATGGCGCCTAAGAAGAAGAGAAAAGTTAAAAGTGCCGAGTACCTCAATACGTTTAGACTGAGGAACTTGGGGCTTCCAGTGATGAATAACTTGCATGACATGTCAAAGGCGACGCGCATTTCAGTTGAAACTCTCAGGTTGCTCATCTATACGGCCGACTTTAGGTACCGGATCTACACGGTTGAGAAGAAGGGCCCGGAGAAACGGATGAGGACCATTTACCAACCTTCGAGAGAGCTGAAAGCCCTCCAGGGTTGGGTCCTGAGGAACATTCTGGATAAACTTTCTTCATCCCCCTTCAGCATCGGGTTCGAGAAACATCAGTCGATACTGAATAATGCAACTCCTCACATCGGAGCGAACTTTATCCTCAACATCGACTTGGAAGATTTCTTTCCGTCTTTGACAGCTAATAAAGTGTTTGGGGTGTTTCACAGTCTTGGTTATAACAGGCTTATTTCATCAGTTCTCACCAAAATCTGCTGTTATAAGAATCTTCTTCCACAGGGGGCACCAAGCTCACCAAAGTTGGCGAACCTGATTTGTTCCAAGCTCGACTACAGGATACAGGGGTATGCAGGAAGTCGCGGACTTATCTACACAAGGTATGCGGACGACCTGACTCTCTCTGCACAATCCATGAAGAAAGTTGTGAAGGCCAGGGACTTCTTGTTCTCTATCATTCCTTCTGAGGGCCTCGTTATCAACAGTAAGAAAACTTGCATCAGCGGGCCGAGGTCACAGAGAAAGGTGACAGGGTTGGTTATCAGTCAAGAGAAAGTGGGAATTGGTAGAGAGAAATACAAGGAAATACGCGCAAAGATCCACCATATATTCTGTGGTAAAAGTAGCGAGATCGAACACGTTAGAGGCTGGCTGTCGTTTATCTTGTCTGTCGACTCAAAGTCCCATAGGCGCCTTATCACGTATATCAGTAAACTTGAAAAGAAATACGGAAAGAATCCACTGAACAAAGCTAAAACTTGAcccgatgagctaaGGATCCCCCGGGgctagctatatcatcaatttatgtattacacataatatcgcactcagtctttcatctacggcaatgtaccagctgatataatcagttattgaaatatttctgaatttaaacttgcatcaataaatttatgtttttgcttggactataatacctgacttgttattttatcaataaatatttaaactatatttctttcaagatTTTTTTGGTGGAGCACGACACTCTCGTCTACTCCAAGAATATCAAAGATACAGTCTCAGAAGACCAAAGGGCTATTGAGACTTTTCAACAAAGGGTAATATCGGGAAACCTCCTCGGATTCCATTGCCCAGCTATCTGTCACTTCATCAAAAGGACAGTAGAAAAGGAAGGTGGCACCTACAAATGCCATCATTGCGATAAAGGAAAGGCTATCGTTCAAGATGCCTCTGCCGACAGTGGTCCCAAAGATGGACCCCCACCCACGAGGAGCATCGTGGAAAAAGAAGACGTTCCAACCACGTCTTCAAAGCAAGTGGATTGATGTGATAACATGGTGGAGCACGACACTCTCGTCTACTCCAAGAATATCAAAGATACAGTCTCAGAAGACCAAAGGGCTATTGAGACTTTTCAACAAAGGGTAATATCGGGAAACCTCCTCGGATTCCATTGCCCAGCTATCTGTCACTTCATCAAAAGGACAGTAGAAAAGGAAGGTGGCACCTACAAATGCCATCATTGCGATAAAGGAAAGGCTATCGTTCAAGATGCCTCTGCCGACAGTGGTCCCAAAGATGGACCCCCACCCACGAGGAGCATCGTGGAAAAAGAAGACGTTCCAACCACGTCTTCAAAGCAAGTGGATTGATGTGATATCTCCACTGACGTAAGGGATGACGCACAATCCCACTATCCTTCGCAAGACCTTCCTCTATATAAGGAAGTTCATTTCATTTGGAGAGGACACGCTGAAATCACCAGTCTCTCTCTACAAATCTATCTCTgggtgcgcatctgatgagtccgtgaggacgaaacgagctagctcgtc**ATGCGCACCCTTAGCGAGAGGTTTATCATTAAGGTCAACCTCTGGATGTTGTTTCGGCATCCTGCATTGAATCTGAGTTACTGTCTGTTTTCCTTCTGTGCTGACTGCGGGGGGATGGGGATCTGGGTGGGGGTTCGCTACGATTCTCTGCTTGATGTGGAGACgaattcaagcttGGGCGGTTGAGATCGGAACTTGTTGGTGATAGATCTGCCTGTATGCTTCGTGTTGCATTAGGAAACCCGTTTCTTCTGACGTAAGGGTGCGCA**CTGCTTGATGTGGAGACGGGgttttagagctagaaatagcaagttaaaataaggctagtccgttatcaacttgaaaaagtggcaccgagtcggtgcGATGGCCGGCATGGTCCCAGCCTCCTCGCTGGCGCCGGCTGGGCAACACCTTCGGGTGGCGAATGGGACTTTATGAAGATGAAGATGAAATATTTGGTGTGTCAAATAAAAAGCTAGCTTGTGTGCTTAAGTTTGTGTTTTTTTCTTGGCTTGTTGTGTTATGAATTTGTGGCTTTTTCTAATATTAAATGAATGTAAGATCTCATTATAATGAATAAACAAATGTTTCTATAATCCATTGTGAATGTTTTGTTGGATCTCTTCGCATATAACTACTGTATGTGCTATGGTATGGACTATGGAATATGATTAAAGATAAGaggtctcggttttagagctagaaatagcaagttaaaataaggctagtccgttatcaacttgaaaaagtggcaccgagtcggtgcttttttgttttagagctagaaatagcaagttaaaataaggctagtccgtttttagcgcgtgcatgcctgcaggtccacaaattcgggtcaaggcggaagccagcgcgccaccccacgtcagcaaatacggaggcgcggggttgacggcgtcacccggtcctaacggcgaccaacaaaccagccagaagaaattacagtaaaaaaaaagtaaattgcactttgatccaccttttattacctaagtctcaatttggatcacccttaaacctatcttttcaatttgggccgggttgtggtttggactaccatgaacaacttttcgtcatgtctaacttccctttcagcaaacatatgaaccatatatagaggagatcggccgtatactagagctgatgtgtttaaggtcgttgattgcacgagaaaaaaaaatccaaatcgcaacaatagcaaatttatctggttcaaagtgaaaagatatgtttaaaggtagtccaaagtaaaacttatagataataaaatgtggtccaaagcgtaattcactcaaaaaaaatcaacgagacgtgtaccaaacggagacaaacggcatcttctcgaaatttcccaaccgctcgctcgcccgcctcgtcttcccggaaaccgcggtggtttcagcgtggcggattctccaagcagacggagacgtcacggcacgggactcctcccaccacccaaccgccataaataccagccccctcatctcctctcctcgcatcagctccacccccgaaaaatttctccccaatctcgcgaggctctcgtcgtcgaatcgaatcctctcgcgtcctcaaggtacgctgcttctcctctcctcgcttcgtttcgattcgatttcggacgggtgaggttgttttgttgctagatccgattggtggttagggttgtcgatgtgattatcgtgagatgtttaggggttgtagatctgatggttgtgatttgggcacggttggttcgataggtggaatcgtggttaggttttgggattggatgttggttctgatgattggggggaatttttacggttagatgaattgttggatgattcgattggggaaatcggtgtagatctgttggggaattgtggaactagtcatgcctgagtgattggtgcgatttgtagcgtgttccatcttgtaggccttgttgcgagcatgttcagatctactgttccgctcttgattgagttattggtgccatgggttggtgcaaacacaggctttaatatgttatatctgttttgtgtttgatgtagatctgtagggtagttcttcttagacatggttcaattatgtagcttgtgcgtttcgatttgatttcatatgttcacagattagataatgatgaactcttttaattaattgtcaatggtaaataggaagtcttgtcgctatatctgtcataatgatctcatgttactatctgccagtaatttatgctaagaactatattagaatatcatgttacaatctgtagtaatatcatgttacaatctgtagttcatctatataatctattgtggtaatttctttttactatctgtgtgaagattattgccactagttcattctacttatttctgaagttcaggatacgtgtgctgttactacctatctgaatacatgtgtgatgtgcctgttactatctttttgaatacatgtatgttctgttggaatatgtttgctgtttgatccgttgttgtgtccttaatcttgtgctagttcttaccctatctgtttggtgattatttcttgcagatagttatcaacaagtttgtacaaaaaagcaggcttcgaaggagatagaaccaattctctaaggaaatacttaaccatggactataaggaccacgacggagactacaaggatcatgatattgattacaaagacgatgacgataagatggccCCAAAGAAGAAGCGGAAGGTCggtatccacggagtcccagcagccgacaagaagtacagcatcggcctggacatcggcaccaactctgtgggctgggccgtgatcaccgacgagtacaaggtgcccagcaagaaattcaaggtgctgggcaacaccgaccggcacagcatcaagaagaacctgatcggagccctgctgttcgacagcggcgaaacagccgaggccacccggctgaagagaaccgccagaagaagatacaccagacggaagaaccggatctgctatctgcaagagatcttcagcaacgagatggccaaggtggacgacagcttcttccacagactggaagagtccttcctggtggaagaggataagaagcacgagcggcaccccatcttcggcaacatcgtggacgaggtggcctaccacgagaagtaccccaccatctaccacctgagaaagaaactggtggacagcaccgacaaggccgacctgcggctgatctatctggccctggcccacatgatcaagttccggggccacttcctgatcgagggcgacctgaaccccgacaacagcgacgtggacaagctgttcatccagctggtgcagacctacaaccagctgttcgaggaaaaccccatcaacgccagcggcgtggacgccaaggccatcctgtctgccagactgagcaagagcagacggctggaaaatctgatcgcccagctgcccggcgagaagaagaatggcctgttcggaaacctgattgccctgagcctgggcctgacccccaacttcaagagcaacttcgacctggccgaggatgccaaactgcagctgagcaaggacacctacgacgacgacctggacaacctgctggcccagatcggcgaccagtacgccgacctgtttctggccgccaagaacctgtccgacgccatcctgctgagcgacatcctgagagtgaacaccgagatcaccaaggcccccctgagcgcctctatgatcaagagatacgacgagcaccaccaggacctgaccctgctgaaagctctcgtgcggcagcagctgcctgagaagtacaaagagattttcttcgaccagagcaagaacggctacgccggctacattgacggcggagccagccaggaagagttctacaagttcatcaagcccatcctggaaaagatggacggcaccgaggaactgctcgtgaagctgaacagagaggacctgctgcggaagcagcggaccttcgacaacggcagcatcccccaccagatccacctgggagagctgcacgccattctgcggcggcaggaagatttttacccattcctgaaggacaaccgggaaaagatcgagaagatcctgaccttccgcatcccctactacgtgggccctctggccaggggaaacagcagattcgcctggatgaccagaaagagcgaggaaaccatcaccccctggaacttcgaggaagtggtggacaagggcgcttccgcccagagcttcatcgagcggatgaccaacttcgataagaacctgcccaacgagaaggtgctgcccaagcacagcctgctgtacgagtacttcaccgtgtataacgagctgaccaaagtgaaatacgtgaccgagggaatgagaaagcccgccttcctgagcggcgagcagaaaaaggccatcgtggacctgctgttcaagaccaaccggaaagtgaccgtgaagcagctgaaagaggactacttcaagaaaatcgagtgcttcgactccgtggaaatctccggcgtggaagatcggttcaacgcctccctgggcacataccacgatctgctgaaaattatcaaggacaaggacttcctggacaatgaggaaaacgaggacattctggaagatatcgtgctgaccctgacactgtttgaggacagagagatgatcgaggaacggctgaaaacctatgcccacctgttcgacgacaaagtgatgaagcagctgaagcggcggagatacaccggctggggcaggctgagccggaagctgatcaacggcatccgggacaagcagtccggcaagacaatcctggatttcctgaagtccgacggcttcgccaacagaaacttcatgcagctgatccacgacgacagcctgacctttaaagaggacatccagaaagcccaggtgtccggccagggcgatagcctgcacgagcacattgccaatctggccggcagccccgccattaagaagggcatcctgcagacagtgaaggtggtggacgagctcgtgaaagtgatgggccggcacaagcccgagaacatcgtgatcgaaatggccagagagaaccagaccacccagaagggacagaagaacagccgcgagagaatgaagcggatcgaagagggcatcaaagagctgggcagccagatcctgaaagaacaccccgtggaaaacacccagctgcagaacgagaagctgtacctgtactacctgcagaatgggcgggatatgtacgtggaccaggaactggacatcaaccggctgtccgactacgatgtggaccatatcgtgcctcagagctttctgaaggacgactccatcgacaacaaggtgctgaccagaagcgacaagaaccggggcaagagcgacaacgtgccctccgaagaggtcgtgaagaagatgaagaactactggcggcagctgctgaacgccaagctgattacccagagaaagttcgacaatctgaccaaggccgagagaggcggcctgagcgaactggataaggccggcttcatcaagagacagctggtggaaacccggcagatcacaaagcacgtggcacagatcctggactcccggatgaacactaagtacgacgagaatgacaagctgatccgggaagtgaaagtgatcaccctgaagtccaagctggtgtccgatttccggaaggatttccagttttacaaagtgcgcgagatcaacaactaccaccacgcccacgacgcctacctgaacgccgtcgtgggaaccgccctgatcaaaaagtaccctaagctggaaagcgagttcgtgtacggcgactacaaggtgtacgacgtgcggaagatgatcgccaagagcgagcaggaaatcggcaaggctaccgccaagtacttcttctacagcaacatcatgaactttttcaagaccgagattaccctggccaacggcgagatccggaagcggcctctgatcgagacaaacggcgaaaccggggagatcgtgtgggataagggccgggattttgccaccgtgcggaaagtgctgagcatgccccaagtgaatatcgtgaaaaagaccgaggtgcagacaggcggcttcagcaaagagtctatcctgcccaagaggaacagcgataagctgatcgccagaaagaaggactgggaccctaagaagtacggcggcttcgacagccccaccgtggcctattctgtgctggtggtggccaaagtggaaaagggcaagtccaagaaactgaagagtgtgaaagagctgctggggatcaccatcatggaaagaagcagcttcgagaagaatcccatcgactttctggaagccaagggctacaaagaagtgaaaaaggacctgatcatcaagctgcctaagtactccctgttcgagctggaaaacggccggaagagaatgctggcctctgccggcgaactgcagaagggaaacgaactggccctgccctccaaatatgtgaacttcctgtacctggccagccactatgagaagctgaagggctcccccgaggataatgagcagaaacagctgtttgtggaacagcacaagcactacctggacgagatcatcgagcagatcagcgagttctccaagagagtgatcctggccgacgctaatctggacaaagtgctgtccgcctacaacaagcaccgggataagcccatcagagagcaggccgagaatatcatccacctgtttaccctgaccaatctgggagcccctgccgccttcaagtactttgacaccaccatcgaccggaagaggtacaccagcaccaaagaggtgctggacgccaccctgatccaccagagcatcaccggcctgtacgagacacggatcgacctgtctcagctgggaggcgacAAAAGGCCGGCGGCCACGAAAAAGGCCGGCCAGGCAAAAAAGAAAAAGtaagaattcgcggccgcactcgagatatctagacccagctttcttgtacaaagtggttgataacagcgactacaaggatgacgatgacaaggcttagagctcgaatttccccgatcgttcaaacatttggcaataaagtttcttaagattgaatcctgttgccggtcttgcgatgattatcatataatttctgttgaattacgttaagcatgtaataattaacatgtaatgcatgacgttatttatgagatgggtttttatgattagagtcccgcaattatacatttaatacgcgatagaaaacaaaatatagcgcgcaaactaggataaattatcgcgcgcggtgtcatctatgttactagatcgggaa

**pK-CRISPEY-ACTIN (**sequences within T-DNA border is shown. Selectable marker cassette is not shown)

| **Colour/other code (sequential)** | **Sequence detail** |
| --- | --- |
| Magenta font | OsUbi10 promoter |
| Green font | SV40 NLS |
| Purple font | Codon optimized EcRT86 |
| Brown | Gene 7 terminator |
| Red font | CaMV35S promoter (enhanced) |
| Dark Yellow | HH ribozyme |
| **Bold:** | Retron including donor seq |
| Underlined: | msr sequence |
| Turquoise: | donor template (intended changes are shown in small letter) |
| Pink: | Actin protospacer |
| Gray: | SpCas9 scaffold |
| Teal: | HDV ribozyme |
| Red: | HSP terminator |
| Blue font | SpCas9 |
| Teal font | Nucleoplasmin NLS |
| Gold font | Nos terminator |

attcgggtcaaggcggaagccagcgcgccaccccacgtcagcaaatacggaggcgcggggttgacggcgtcacccggtcctaacggcgaccaacaaaccagccagaagaaattacagtaaaaaaaaagtaaattgcactttgatccaccttttattacctaagtctcaatttggatcacccttaaacctatcttttcaatttgggccgggttgtggtttggactaccatgaacaacttttcgtcatgtctaacttccctttcagcaaacatatgaaccatatatagaggagatcggccgtatactagagctgatgtgtttaaggtcgttgattgcacgagaaaaaaaaatccaaatcgcaacaatagcaaatttatctggttcaaagtgaaaagatatgtttaaaggtagtccaaagtaaaacttatagataataaaatgtggtccaaagcgtaattcactcaaaaaaaatcaacgagacgtgtaccaaacggagacaaacggcatcttctcgaaatttcccaaccgctcgctcgcccgcctcgtcttcccggaaaccgcggtggtttcagcgtggcggattctccaagcagacggagacgtcacggcacgggactcctcccaccacccaaccgccataaataccagccccctcatctcctctcctcgcatcagctccacccccgaaaaatttctccccaatctcgcgaggctctcgtcgtcgaatcgaatcctctcgcgtcctcaaggtacgctgcttctcctctcctcgcttcgtttcgattcgatttcggacgggtgaggttgttttgttgctagatccgattggtggttagggttgtcgatgtgattatcgtgagatgtttaggggttgtagatctgatggttgtgatttgggcacggttggttcgataggtggaatcgtggttaggttttgggattggatgttggttctgatgattggggggaatttttacggttagatgaattgttggatgattcgattggggaaatcggtgtagatctgttggggaattgtggaactagtcatgcctgagtgattggtgcgatttgtagcgtgttccatcttgtaggccttgttgcgagcatgttcagatctactgttccgctcttgattgagttattggtgccatgggttggtgcaaacacaggctttaatatgttatatctgttttgtgtttgatgtagatctgtagggtagttcttcttagacatggttcaattatgtagcttgtgcgtttcgatttgatttcatatgttcacagattagataatgatgaactcttttaattaattgtcaatggtaaataggaagtcttgtcgctatatctgtcataatgatctcatgttactatctgccagtaatttatgctaagaactatattagaatatcatgttacaatctgtagtaatatcatgttacaatctgtagttcatctatataatctattgtggtaatttctttttactatctgtgtgaagattattgccactagttcattctacttatttctgaagttcaggatacgtgtgctgttactacctatctgaatacatgtgtgatgtgcctgttactatctttttgaatacatgtatgttctgttggaatatgtttgctgtttgatccgttgttgtgtccttaatcttgtgctagttcttaccctatctgtttggtgattatttcttgcagatagttatcaacaagtttgtacCGAGCTCGGATCCACACCATGGCGCCTAAGAAGAAGAGAAAAGTTAAAAGTGCCGAGTACCTCAATACGTTTAGACTGAGGAACTTGGGGCTTCCAGTGATGAATAACTTGCATGACATGTCAAAGGCGACGCGCATTTCAGTTGAAACTCTCAGGTTGCTCATCTATACGGCCGACTTTAGGTACCGGATCTACACGGTTGAGAAGAAGGGCCCGGAGAAACGGATGAGGACCATTTACCAACCTTCGAGAGAGCTGAAAGCCCTCCAGGGTTGGGTCCTGAGGAACATTCTGGATAAACTTTCTTCATCCCCCTTCAGCATCGGGTTCGAGAAACATCAGTCGATACTGAATAATGCAACTCCTCACATCGGAGCGAACTTTATCCTCAACATCGACTTGGAAGATTTCTTTCCGTCTTTGACAGCTAATAAAGTGTTTGGGGTGTTTCACAGTCTTGGTTATAACAGGCTTATTTCATCAGTTCTCACCAAAATCTGCTGTTATAAGAATCTTCTTCCACAGGGGGCACCAAGCTCACCAAAGTTGGCGAACCTGATTTGTTCCAAGCTCGACTACAGGATACAGGGGTATGCAGGAAGTCGCGGACTTATCTACACAAGGTATGCGGACGACCTGACTCTCTCTGCACAATCCATGAAGAAAGTTGTGAAGGCCAGGGACTTCTTGTTCTCTATCATTCCTTCTGAGGGCCTCGTTATCAACAGTAAGAAAACTTGCATCAGCGGGCCGAGGTCACAGAGAAAGGTGACAGGGTTGGTTATCAGTCAAGAGAAAGTGGGAATTGGTAGAGAGAAATACAAGGAAATACGCGCAAAGATCCACCATATATTCTGTGGTAAAAGTAGCGAGATCGAACACGTTAGAGGCTGGCTGTCGTTTATCTTGTCTGTCGACTCAAAGTCCCATAGGCGCCTTATCACGTATATCAGTAAACTTGAAAAGAAATACGGAAAGAATCCACTGAACAAAGCTAAAACTTGAcccgatgagctaaGGATCCCCCGGGgctagctatatcatcaatttatgtattacacataatatcgcactcagtctttcatctacggcaatgtaccagctgatataatcagttattgaaatatttctgaatttaaacttgcatcaataaatttatgtttttgcttggactataatacctgacttgttattttatcaataaatatttaaactatatttctttcaagatTTTTTTGGTGGAGCACGACACTCTCGTCTACTCCAAGAATATCAAAGATACAGTCTCAGAAGACCAAAGGGCTATTGAGACTTTTCAACAAAGGGTAATATCGGGAAACCTCCTCGGATTCCATTGCCCAGCTATCTGTCACTTCATCAAAAGGACAGTAGAAAAGGAAGGTGGCACCTACAAATGCCATCATTGCGATAAAGGAAAGGCTATCGTTCAAGATGCCTCTGCCGACAGTGGTCCCAAAGATGGACCCCCACCCACGAGGAGCATCGTGGAAAAAGAAGACGTTCCAACCACGTCTTCAAAGCAAGTGGATTGATGTGATAACATGGTGGAGCACGACACTCTCGTCTACTCCAAGAATATCAAAGATACAGTCTCAGAAGACCAAAGGGCTATTGAGACTTTTCAACAAAGGGTAATATCGGGAAACCTCCTCGGATTCCATTGCCCAGCTATCTGTCACTTCATCAAAAGGACAGTAGAAAAGGAAGGTGGCACCTACAAATGCCATCATTGCGATAAAGGAAAGGCTATCGTTCAAGATGCCTCTGCCGACAGTGGTCCCAAAGATGGACCCCCACCCACGAGGAGCATCGTGGAAAAAGAAGACGTTCCAACCACGTCTTCAAAGCAAGTGGATTGATGTGATATCTCCACTGACGTAAGGGATGACGCACAATCCCACTATCCTTCGCAAGACCTTCCTCTATATAAGGAAGTTCATTTCATTTGGAGAGGACACGCTGAAATCACCAGTCTCTCTCTACAAATCTATCTCTGGGTGCGCATctgatgagtccgtgaggacgaaacgagctagctcgtc**ATGCGCACCCTTAGCGAGAGGTTTATCATTAAGGTCAACCTCTGGATGTTGTTTCGGCATCCTGCATTGAATCTGAGTTACTGTCTGTTTTCCTGCTTACAATGTTGCTTGCCGTTGCAGATGTGGATTGCCAAGGCTGAGTACGACGAGTCTGGCCCATCCATTGTGgaattcCACAGGAAATGCTTCTAATTCTTCGGACCCAAGAATGCTAAGCCAAGAGGAGCTGTTATCGCCGTCCTCCAGGAAACCCGTTTCTTCTGACGTAAGGGTGCGCA**CTGGCCCATCCATTGTGCACgttttagagctagaaatagcaagttaaaataaggctagtccgttatcaacttgaaaaagtggcaccgagtcggtgcGATGGCCGGCATGGTCCCAGCCTCCTCGCTGGCGCCGGCTGGGCAACACCTTCGGGTGGCGAATGGGACTTTATGAAGATGAAGATGAAATATTTGGTGTGTCAAATAAAAAGCTAGCTTGTGTGCTTAAGTTTGTGTTTTTTTCTTGGCTTGTTGTGTTATGAATTTGTGGCTTTTTCTAATATTAAATGAATGTAAGATCTCATTATAATGAATAAACAAATGTTTCTATAATCCATTGTGAATGTTTTGTTGGATCTCTTCGCATATAACTACTGTATGTGCTATGGTATGGACTATGGAATATGATTAAAGATAAGaggtctcggttttagagctagaaatagcaagttaaaataaggctagtccgttatcaacttgaaaaagtggcaccgagtcggtgcttttttgttttagagctagaaatagcaagttaaaataaggctagtccgtttttagcgcgtgcatgcctgcaggtccacaaattcgggtcaaggcggaagccagcgcgccaccccacgtcagcaaatacggaggcgcggggttgacggcgtcacccggtcctaacggcgaccaacaaaccagccagaagaaattacagtaaaaaaaaagtaaattgcactttgatccaccttttattacctaagtctcaatttggatcacccttaaacctatcttttcaatttgggccgggttgtggtttggactaccatgaacaacttttcgtcatgtctaacttccctttcagcaaacatatgaaccatatatagaggagatcggccgtatactagagctgatgtgtttaaggtcgttgattgcacgagaaaaaaaaatccaaatcgcaacaatagcaaatttatctggttcaaagtgaaaagatatgtttaaaggtagtccaaagtaaaacttatagataataaaatgtggtccaaagcgtaattcactcaaaaaaaatcaacgagacgtgtaccaaacggagacaaacggcatcttctcgaaatttcccaaccgctcgctcgcccgcctcgtcttcccggaaaccgcggtggtttcagcgtggcggattctccaagcagacggagacgtcacggcacgggactcctcccaccacccaaccgccataaataccagccccctcatctcctctcctcgcatcagctccacccccgaaaaatttctccccaatctcgcgaggctctcgtcgtcgaatcgaatcctctcgcgtcctcaaggtacgctgcttctcctctcctcgcttcgtttcgattcgatttcggacgggtgaggttgttttgttgctagatccgattggtggttagggttgtcgatgtgattatcgtgagatgtttaggggttgtagatctgatggttgtgatttgggcacggttggttcgataggtggaatcgtggttaggttttgggattggatgttggttctgatgattggggggaatttttacggttagatgaattgttggatgattcgattggggaaatcggtgtagatctgttggggaattgtggaactagtcatgcctgagtgattggtgcgatttgtagcgtgttccatcttgtaggccttgttgcgagcatgttcagatctactgttccgctcttgattgagttattggtgccatgggttggtgcaaacacaggctttaatatgttatatctgttttgtgtttgatgtagatctgtagggtagttcttcttagacatggttcaattatgtagcttgtgcgtttcgatttgatttcatatgttcacagattagataatgatgaactcttttaattaattgtcaatggtaaataggaagtcttgtcgctatatctgtcataatgatctcatgttactatctgccagtaatttatgctaagaactatattagaatatcatgttacaatctgtagtaatatcatgttacaatctgtagttcatctatataatctattgtggtaatttctttttactatctgtgtgaagattattgccactagttcattctacttatttctgaagttcaggatacgtgtgctgttactacctatctgaatacatgtgtgatgtgcctgttactatctttttgaatacatgtatgttctgttggaatatgtttgctgtttgatccgttgttgtgtccttaatcttgtgctagttcttaccctatctgtttggtgattatttcttgcagatagttatcaacaagtttgtacaaaaaagcaggcttcgaaggagatagaaccaattctctaaggaaatacttaaccatggactataaggaccacgacggagactacaaggatcatgatattgattacaaagacgatgacgataagatggccCCAAAGAAGAAGCGGAAGGTCggtatccacggagtcccagcagccgacaagaagtacagcatcggcctggacatcggcaccaactctgtgggctgggccgtgatcaccgacgagtacaaggtgcccagcaagaaattcaaggtgctgggcaacaccgaccggcacagcatcaagaagaacctgatcggagccctgctgttcgacagcggcgaaacagccgaggccacccggctgaagagaaccgccagaagaagatacaccagacggaagaaccggatctgctatctgcaagagatcttcagcaacgagatggccaaggtggacgacagcttcttccacagactggaagagtccttcctggtggaagaggataagaagcacgagcggcaccccatcttcggcaacatcgtggacgaggtggcctaccacgagaagtaccccaccatctaccacctgagaaagaaactggtggacagcaccgacaaggccgacctgcggctgatctatctggccctggcccacatgatcaagttccggggccacttcctgatcgagggcgacctgaaccccgacaacagcgacgtggacaagctgttcatccagctggtgcagacctacaaccagctgttcgaggaaaaccccatcaacgccagcggcgtggacgccaaggccatcctgtctgccagactgagcaagagcagacggctggaaaatctgatcgcccagctgcccggcgagaagaagaatggcctgttcggaaacctgattgccctgagcctgggcctgacccccaacttcaagagcaacttcgacctggccgaggatgccaaactgcagctgagcaaggacacctacgacgacgacctggacaacctgctggcccagatcggcgaccagtacgccgacctgtttctggccgccaagaacctgtccgacgccatcctgctgagcgacatcctgagagtgaacaccgagatcaccaaggcccccctgagcgcctctatgatcaagagatacgacgagcaccaccaggacctgaccctgctgaaagctctcgtgcggcagcagctgcctgagaagtacaaagagattttcttcgaccagagcaagaacggctacgccggctacattgacggcggagccagccaggaagagttctacaagttcatcaagcccatcctggaaaagatggacggcaccgaggaactgctcgtgaagctgaacagagaggacctgctgcggaagcagcggaccttcgacaacggcagcatcccccaccagatccacctgggagagctgcacgccattctgcggcggcaggaagatttttacccattcctgaaggacaaccgggaaaagatcgagaagatcctgaccttccgcatcccctactacgtgggccctctggccaggggaaacagcagattcgcctggatgaccagaaagagcgaggaaaccatcaccccctggaacttcgaggaagtggtggacaagggcgcttccgcccagagcttcatcgagcggatgaccaacttcgataagaacctgcccaacgagaaggtgctgcccaagcacagcctgctgtacgagtacttcaccgtgtataacgagctgaccaaagtgaaatacgtgaccgagggaatgagaaagcccgccttcctgagcggcgagcagaaaaaggccatcgtggacctgctgttcaagaccaaccggaaagtgaccgtgaagcagctgaaagaggactacttcaagaaaatcgagtgcttcgactccgtggaaatctccggcgtggaagatcggttcaacgcctccctgggcacataccacgatctgctgaaaattatcaaggacaaggacttcctggacaatgaggaaaacgaggacattctggaagatatcgtgctgaccctgacactgtttgaggacagagagatgatcgaggaacggctgaaaacctatgcccacctgttcgacgacaaagtgatgaagcagctgaagcggcggagatacaccggctggggcaggctgagccggaagctgatcaacggcatccgggacaagcagtccggcaagacaatcctggatttcctgaagtccgacggcttcgccaacagaaacttcatgcagctgatccacgacgacagcctgacctttaaagaggacatccagaaagcccaggtgtccggccagggcgatagcctgcacgagcacattgccaatctggccggcagccccgccattaagaagggcatcctgcagacagtgaaggtggtggacgagctcgtgaaagtgatgggccggcacaagcccgagaacatcgtgatcgaaatggccagagagaaccagaccacccagaagggacagaagaacagccgcgagagaatgaagcggatcgaagagggcatcaaagagctgggcagccagatcctgaaagaacaccccgtggaaaacacccagctgcagaacgagaagctgtacctgtactacctgcagaatgggcgggatatgtacgtggaccaggaactggacatcaaccggctgtccgactacgatgtggaccatatcgtgcctcagagctttctgaaggacgactccatcgacaacaaggtgctgaccagaagcgacaagaaccggggcaagagcgacaacgtgccctccgaagaggtcgtgaagaagatgaagaactactggcggcagctgctgaacgccaagctgattacccagagaaagttcgacaatctgaccaaggccgagagaggcggcctgagcgaactggataaggccggcttcatcaagagacagctggtggaaacccggcagatcacaaagcacgtggcacagatcctggactcccggatgaacactaagtacgacgagaatgacaagctgatccgggaagtgaaagtgatcaccctgaagtccaagctggtgtccgatttccggaaggatttccagttttacaaagtgcgcgagatcaacaactaccaccacgcccacgacgcctacctgaacgccgtcgtgggaaccgccctgatcaaaaagtaccctaagctggaaagcgagttcgtgtacggcgactacaaggtgtacgacgtgcggaagatgatcgccaagagcgagcaggaaatcggcaaggctaccgccaagtacttcttctacagcaacatcatgaactttttcaagaccgagattaccctggccaacggcgagatccggaagcggcctctgatcgagacaaacggcgaaaccggggagatcgtgtgggataagggccgggattttgccaccgtgcggaaagtgctgagcatgccccaagtgaatatcgtgaaaaagaccgaggtgcagacaggcggcttcagcaaagagtctatcctgcccaagaggaacagcgataagctgatcgccagaaagaaggactgggaccctaagaagtacggcggcttcgacagccccaccgtggcctattctgtgctggtggtggccaaagtggaaaagggcaagtccaagaaactgaagagtgtgaaagagctgctggggatcaccatcatggaaagaagcagcttcgagaagaatcccatcgactttctggaagccaagggctacaaagaagtgaaaaaggacctgatcatcaagctgcctaagtactccctgttcgagctggaaaacggccggaagagaatgctggcctctgccggcgaactgcagaagggaaacgaactggccctgccctccaaatatgtgaacttcctgtacctggccagccactatgagaagctgaagggctcccccgaggataatgagcagaaacagctgtttgtggaacagcacaagcactacctggacgagatcatcgagcagatcagcgagttctccaagagagtgatcctggccgacgctaatctggacaaagtgctgtccgcctacaacaagcaccgggataagcccatcagagagcaggccgagaatatcatccacctgtttaccctgaccaatctgggagcccctgccgccttcaagtactttgacaccaccatcgaccggaagaggtacaccagcaccaaagaggtgctggacgccaccctgatccaccagagcatcaccggcctgtacgagacacggatcgacctgtctcagctgggaggcgacAAAAGGCCGGCGGCCACGAAAAAGGCCGGCCAGGCAAAAAAGAAAAAGtaagaattcgcggccgcactcgagatatctagacccagctttcttgtacaaagtggttgataacagcgactacaaggatgacgatgacaaggcttagagctcgaatttccccgatcgttcaaacatttggcaataaagtttcttaagattgaatcctgttgccggtcttgcgatgattatcatataatttctgttgaattacgttaagcatgtaataattaacatgtaatgcatgacgttatttatgagatgggtttttatgattagagtcccgcaattatacatttaatacgcgatagaaaacaaaatatagcgcgcaaactaggataaattatcgcgcgcggtgtcatctatgttactagatcgggaa

**pCgAPP**

| **Colour/other code (sequential)** | **Sequence detail** |
| --- | --- |
| Dashed underline | OsU3 promoter |
| Green: | tRNA |
| Pink: | *ALS* protospacer |
| Gray: | SpCas9 scaffold |
| Turquoise: | *ALS* donor template (intended changes are shown in small letter) |
| Red: | *Pita* protospacer |
| Teal: | *Pita* donor template |
| Yellow: | *Ptr* protospacer |
| Dark Yellow: | *Ptr* donor template (intended changes are shown in small letter. 12 bp deletion is not shown). |
| Magenta font | OsUbi10 promoter |
| Green font | SV40 NLS |
| Blue font | SpCas9 |
| Teal font | Nucleoplasmin NLS |
| Gold font | Nos terminator |

agcttAAGGAATCTTTAAACATACGAACAGATCACTTAAAGTTCTTCTGAAGCAACTTAAAGTTATCAGGCATGCATGGATCTTGGAGGAATCAGATGTGCAGTCAGGGACCATAGCACAAGACAGGCGTCTTCTACTGGTGCTACCAGCAAATGCTGGAAGCCGGGAACACTGGGTACGTTGGAAACCACGTGATGTGAAGAAGTAAGATAAACTGTAGGAGAAAAGCATTTCGTAGTGGGCCATGAAGCCTTTCAGGAcatgtattgcagtatgggccggcccattacgcaattggacgacaacaaagactagtattagtaccacctcggctatccacatagatcaaagctgatttaaaagagttgtgcagatgatccgtGGCaacaaagcaccagtggtctagtggtagaatagtaccctgccacggtacagacccgggttcgattcccggctggtgcaGGGTATGGTGGTGCAATGGGgttttagagctagaaatagcaagttaaaataaggctagtccgttatcaacttgaaaaagtggcaccgagtcggtgcCCCTTAGCAATAGTCACAAAATCTGGATATATCTCGCTCTCACATTCCGGGTTGCCCAAGTATGTATGCGCCCTATTCGCCTTGTAAAACCTATCtTCCaATTGgACgACCATACCCAAATGTTGGTTGTTCAACACCATCACCTTCACAGGGAGGTTCTCAATGCGGATCAATGCCAGCTCCTGAATGTTCATGAGGAAaacaaagcaccagtggtctagtggtagaatagtaccctgccacggtacagacccgggttcgattcccggctggtgcaTCAGGTTGAAGATGCATAGAgttttagagctagaaatagcaagttaaaataaggctagtccgttatcaacttgaaaaagtggcaccgagtcggtgcGATGATCACGGGTATGGATTTTTCATTCTATTCCCAGGTTACAACTTACAAGGATTATTGAGCTTCTTTCTTTCTCTGCCaTGGCTTCTATCTTTACCTgCgATGCActTgCAACCTGACTTGATGATTGTTTGAAACCAATTTTAATGGAAGTTAAATGTTATTGTTGTGACCCTGAATCAGGTTTTGTATGCTACCGGaacaaagcaccagtggtctagtggtagaatagtaccctgccacggtacagacccgggttcgattcccggctggtgcaAAAAACCAAAACCAGCTGCCgttttagagctagaaatagcaagttaaaataaggctagtccgttatcaacttgaaaaagtggcaccgagtcggtgcCGGAGAAAGTACAAATTAGGGTGTTATCAAGAGATACAACACGCGTTGGGATCTTCCTCGaCCAAAAACATAtAGTGGGGCGGGGgGTATTTCGCAGgCAtAAACTCGTACgGTTCCGGCtTGGCAGgTGGTTCAGGCATAAATTCGATGCGATGAACTTTTACAGCTTCGTTGATGAGGTCTGCGAAGGTCATGCGAGGCTTCGTTGTCTGGTCGCAGATACCAGCGGCGATGTTGTTGAACCGAGCAGTTTTTTTTTTgttttagagctagaaatagcaagttaaaataaggctagtccgttatcaacttgaaaaagtggcaccgagtcggtgcttttttgttttagagctagaaatagcaagttaaaataaggctagtccgtttttagcgcgtgcatgcctgcaggtccacaaattcgggtcaaggcggaagccagcgcgccaccccacgtcagcaaatacggaggcgcggggttgacggcgtcacccggtcctaacggcgaccaacaaaccagccagaagaaattacagtaaaaaaaaagtaaattgcactttgatccaccttttattacctaagtctcaatttggatcacccttaaacctatcttttcaatttgggccgggttgtggtttggactaccatgaacaacttttcgtcatgtctaacttccctttcagcaaacatatgaaccatatatagaggagatcggccgtatactagagctgatgtgtttaaggtcgttgattgcacgagaaaaaaaaatccaaatcgcaacaatagcaaatttatctggttcaaagtgaaaagatatgtttaaaggtagtccaaagtaaaacttatagataataaaatgtggtccaaagcgtaattcactcaaaaaaaatcaacgagacgtgtaccaaacggagacaaacggcatcttctcgaaatttcccaaccgctcgctcgcccgcctcgtcttcccggaaaccgcggtggtttcagcgtggcggattctccaagcagacggagacgtcacggcacgggactcctcccaccacccaaccgccataaataccagccccctcatctcctctcctcgcatcagctccacccccgaaaaatttctccccaatctcgcgaggctctcgtcgtcgaatcgaatcctctcgcgtcctcaaggtacgctgcttctcctctcctcgcttcgtttcgattcgatttcggacgggtgaggttgttttgttgctagatccgattggtggttagggttgtcgatgtgattatcgtgagatgtttaggggttgtagatctgatggttgtgatttgggcacggttggttcgataggtggaatcgtggttaggttttgggattggatgttggttctgatgattggggggaatttttacggttagatgaattgttggatgattcgattggggaaatcggtgtagatctgttggggaattgtggaactagtcatgcctgagtgattggtgcgatttgtagcgtgttccatcttgtaggccttgttgcgagcatgttcagatctactgttccgctcttgattgagttattggtgccatgggttggtgcaaacacaggctttaatatgttatatctgttttgtgtttgatgtagatctgtagggtagttcttcttagacatggttcaattatgtagcttgtgcgtttcgatttgatttcatatgttcacagattagataatgatgaactcttttaattaattgtcaatggtaaataggaagtcttgtcgctatatctgtcataatgatctcatgttactatctgccagtaatttatgctaagaactatattagaatatcatgttacaatctgtagtaatatcatgttacaatctgtagttcatctatataatctattgtggtaatttctttttactatctgtgtgaagattattgccactagttcattctacttatttctgaagttcaggatacgtgtgctgttactacctatctgaatacatgtgtgatgtgcctgttactatctttttgaatacatgtatgttctgttggaatatgtttgctgtttgatccgttgttgtgtccttaatcttgtgctagttcttaccctatctgtttggtgattatttcttgcagatagttatcaacaagtttgtacaaaaaagcaggcttcgaaggagatagaaccaattctctaaggaaatacttaaccatggactataaggaccacgacggagactacaaggatcatgatattgattacaaagacgatgacgataagatggccCCAAAGAAGAAGCGGAAGGTCggtatccacggagtcccagcagccgacaagaagtacagcatcggcctggacatcggcaccaactctgtgggctgggccgtgatcaccgacgagtacaaggtgcccagcaagaaattcaaggtgctgggcaacaccgaccggcacagcatcaagaagaacctgatcggagccctgctgttcgacagcggcgaaacagccgaggccacccggctgaagagaaccgccagaagaagatacaccagacggaagaaccggatctgctatctgcaagagatcttcagcaacgagatggccaaggtggacgacagcttcttccacagactggaagagtccttcctggtggaagaggataagaagcacgagcggcaccccatcttcggcaacatcgtggacgaggtggcctaccacgagaagtaccccaccatctaccacctgagaaagaaactggtggacagcaccgacaaggccgacctgcggctgatctatctggccctggcccacatgatcaagttccggggccacttcctgatcgagggcgacctgaaccccgacaacagcgacgtggacaagctgttcatccagctggtgcagacctacaaccagctgttcgaggaaaaccccatcaacgccagcggcgtggacgccaaggccatcctgtctgccagactgagcaagagcagacggctggaaaatctgatcgcccagctgcccggcgagaagaagaatggcctgttcggaaacctgattgccctgagcctgggcctgacccccaacttcaagagcaacttcgacctggccgaggatgccaaactgcagctgagcaaggacacctacgacgacgacctggacaacctgctggcccagatcggcgaccagtacgccgacctgtttctggccgccaagaacctgtccgacgccatcctgctgagcgacatcctgagagtgaacaccgagatcaccaaggcccccctgagcgcctctatgatcaagagatacgacgagcaccaccaggacctgaccctgctgaaagctctcgtgcggcagcagctgcctgagaagtacaaagagattttcttcgaccagagcaagaacggctacgccggctacattgacggcggagccagccaggaagagttctacaagttcatcaagcccatcctggaaaagatggacggcaccgaggaactgctcgtgaagctgaacagagaggacctgctgcggaagcagcggaccttcgacaacggcagcatcccccaccagatccacctgggagagctgcacgccattctgcggcggcaggaagatttttacccattcctgaaggacaaccgggaaaagatcgagaagatcctgaccttccgcatcccctactacgtgggccctctggccaggggaaacagcagattcgcctggatgaccagaaagagcgaggaaaccatcaccccctggaacttcgaggaagtggtggacaagggcgcttccgcccagagcttcatcgagcggatgaccaacttcgataagaacctgcccaacgagaaggtgctgcccaagcacagcctgctgtacgagtacttcaccgtgtataacgagctgaccaaagtgaaatacgtgaccgagggaatgagaaagcccgccttcctgagcggcgagcagaaaaaggccatcgtggacctgctgttcaagaccaaccggaaagtgaccgtgaagcagctgaaagaggactacttcaagaaaatcgagtgcttcgactccgtggaaatctccggcgtggaagatcggttcaacgcctccctgggcacataccacgatctgctgaaaattatcaaggacaaggacttcctggacaatgaggaaaacgaggacattctggaagatatcgtgctgaccctgacactgtttgaggacagagagatgatcgaggaacggctgaaaacctatgcccacctgttcgacgacaaagtgatgaagcagctgaagcggcggagatacaccggctggggcaggctgagccggaagctgatcaacggcatccgggacaagcagtccggcaagacaatcctggatttcctgaagtccgacggcttcgccaacagaaacttcatgcagctgatccacgacgacagcctgacctttaaagaggacatccagaaagcccaggtgtccggccagggcgatagcctgcacgagcacattgccaatctggccggcagccccgccattaagaagggcatcctgcagacagtgaaggtggtggacgagctcgtgaaagtgatgggccggcacaagcccgagaacatcgtgatcgaaatggccagagagaaccagaccacccagaagggacagaagaacagccgcgagagaatgaagcggatcgaagagggcatcaaagagctgggcagccagatcctgaaagaacaccccgtggaaaacacccagctgcagaacgagaagctgtacctgtactacctgcagaatgggcgggatatgtacgtggaccaggaactggacatcaaccggctgtccgactacgatgtggaccatatcgtgcctcagagctttctgaaggacgactccatcgacaacaaggtgctgaccagaagcgacaagaaccggggcaagagcgacaacgtgccctccgaagaggtcgtgaagaagatgaagaactactggcggcagctgctgaacgccaagctgattacccagagaaagttcgacaatctgaccaaggccgagagaggcggcctgagcgaactggataaggccggcttcatcaagagacagctggtggaaacccggcagatcacaaagcacgtggcacagatcctggactcccggatgaacactaagtacgacgagaatgacaagctgatccgggaagtgaaagtgatcaccctgaagtccaagctggtgtccgatttccggaaggatttccagttttacaaagtgcgcgagatcaacaactaccaccacgcccacgacgcctacctgaacgccgtcgtgggaaccgccctgatcaaaaagtaccctaagctggaaagcgagttcgtgtacggcgactacaaggtgtacgacgtgcggaagatgatcgccaagagcgagcaggaaatcggcaaggctaccgccaagtacttcttctacagcaacatcatgaactttttcaagaccgagattaccctggccaacggcgagatccggaagcggcctctgatcgagacaaacggcgaaaccggggagatcgtgtgggataagggccgggattttgccaccgtgcggaaagtgctgagcatgccccaagtgaatatcgtgaaaaagaccgaggtgcagacaggcggcttcagcaaagagtctatcctgcccaagaggaacagcgataagctgatcgccagaaagaaggactgggaccctaagaagtacggcggcttcgacagccccaccgtggcctattctgtgctggtggtggccaaagtggaaaagggcaagtccaagaaactgaagagtgtgaaagagctgctggggatcaccatcatggaaagaagcagcttcgagaagaatcccatcgactttctggaagccaagggctacaaagaagtgaaaaaggacctgatcatcaagctgcctaagtactccctgttcgagctggaaaacggccggaagagaatgctggcctctgccggcgaactgcagaagggaaacgaactggccctgccctccaaatatgtgaacttcctgtacctggccagccactatgagaagctgaagggctcccccgaggataatgagcagaaacagctgtttgtggaacagcacaagcactacctggacgagatcatcgagcagatcagcgagttctccaagagagtgatcctggccgacgctaatctggacaaagtgctgtccgcctacaacaagcaccgggataagcccatcagagagcaggccgagaatatcatccacctgtttaccctgaccaatctgggagcccctgccgccttcaagtactttgacaccaccatcgaccggaagaggtacaccagcaccaaagaggtgctggacgccaccctgatccaccagagcatcaccggcctgtacgagacacggatcgacctgtctcagctgggaggcgacAAAAGGCCGGCGGCCACGAAAAAGGCCGGCCAGGCAAAAAAGAAAAAGtaagaattcgcggccgcactcgagatatctagacccagctttcttgtacaaagtggttgataacagcgactacaaggatgacgatgacaaggcttagagctcgaatttccccgatcgttcaaacatttggcaataaagtttcttaagattgaatcctgttgccggtcttgcgatgattatcatataatttctgttgaattacgttaagcatgtaataattaacatgtaatgcatgacgttatttatgagatgggtttttatgattagagtcccgcaattatacatttaatacgcgatagaaaacaaaatatagcgcgcaaactaggataaattatcgcgcgcggtgtcatctatgttactagatcgggaatt
